# Genome-wide modeling of DNA replication in space and time confirms the emergence of replication specific patterns in vivo in eukaryotes

**DOI:** 10.1186/s13059-025-03872-4

**Published:** 2025-12-22

**Authors:** Dario D’Asaro, Jean-Michel Arbona, Vinciane Piveteau, Aurèle Piazza, Cédric Vaillant, Daniel Jost

**Affiliations:** 1 Laboratoire de Biologie et Modélisation de la Cellule, École Normale Supérieure de Lyon, CNRS, UMR5239, Inserm U1293, Université Claude Bernard Lyon 1, 46 Allée d’Italie, 69007 Lyon, France; 2 École Normale Supérieure de Lyon, CNRS, Laboratoire de Physique, 46 Allée d’Italie, Lyon, 69007 France; 3 Present Address: Sainsbury Laboratory, University of Cambridge, Cambridge, CB2 1LR UK; 4 Present Address: Aix Marseille University, CNRS, IBDM, Turing Centre for Living Systems, Parc Scientifique de Luminy, 13288 Marseille, France

## Abstract

**Background:**

Although significant progress has been made in our understanding of DNA replication and spatial chromosome organization in eukaryotes, how they interplay remains elusive. In particular, from the local structure of two diverging sister-forks to the higher-level organization of the replication machinery into nuclear domains, the mechanistic details of chromatin duplication in the 3D nuclear space remain debated.

**Results:**

In this study, we use a computational model of the *Saccharomyces cerevisiae* genome to explore how replication influences chromatin folding. By integrating both a realistic description of the genome 3D architecture and 1D replication timing, simulations reveal that the colocalization of sister-forks produces a characteristic “fountain” pattern around early origins of replication. We confirm the presence of similar features in vivo in early S-phase with new Hi-C data in various conditions, showing that it is replication-dependent and cohesin-independent. At a larger scale, we show that the 3D genome leads to forks being highly enriched at one pole of the nucleus in early S-phase, before later redistributing more homogeneously, and may favor the higher-order clustering of forks into Replication Foci, as observed in earlier microscopy experiments. Additionally, replication causes temporary chromatin slowdown and reduced mobility due to fork passage and sister chromatid intertwining.

**Conclusions:**

Overall, our model offers new insights into the spatial and dynamic organization of chromatin during replication in eukaryotes.

**Supplementary Information:**

The online version contains supplementary material available at 10.1186/s13059-025-03872-4.

## Background

Understanding how living cells duplicate, functionally reorganize and transmit their genomic information represents a fundamental challenge in molecular biology. In eukaryotes, such a process initiates at multiple genomic positions, the so-called origins of replication, and proceeds to the copy of the genome. Origin firing is hierarchical and finely regulated, creating distinctive and heterogeneous patterns of replication timing along the linear genome, known as the “replication timing program” [1, 2]. Although traditionally investigated as a unidimensional process along the genome, advances in 3D genomics [3–6] highlighted a strong correlation between the tridimensional genome organization and the unidimensional replication dynamics that occurs within the crowded environment of the nucleus. In particular, in-depth analysis of genomics data in metazoan have suggested a significant overlap between genomic regions that exhibit coordinated replication and known features of the 3D genome organization such as compartments, TADs and of chromatin regulatory landscape such as epigenomic marks and transcriptional activity [7–14]. For example, the euchromatic A-compartment is enriched in early replicating regions, while the heterochromatic B-compartment in late-replicating ones [9, 14]. In mammals, TAD and replication-unit boundaries largely coincide [7] and cohesin-mediated loops are associated with early replication initiation zones [13, 15]. However, establishing causal relationships between these phenomena remains challenging, and our understanding of these crosstalks is limited. While, on the one hand, the local chromatin architecture and accessibility likely regulate the Replication Timing Program [7, 8, 10–15], on the other hand, the active progression of the replication forks may also influence the spatial folding of chromosomes. For this reason, over the past decades, significant efforts have been devoted to describe the organization of replication forks in the 3D nuclear space [16–22].

During DNA replication, a first layer of organization lies in the 3D architecture of a single replication bubble (or replicon), a scale also relevant for prokaryotic genomes [23]. Currently, two models have been proposed to describe the relative 3D organization of the two diverging forks emanating from the same firing event, the so-called sister-forks or sister-replisomes [24]. The first model proposes that sister-forks remain in close spatial proximity as they progress in opposite directions along the genome, resulting in the effective extrusion of the two Sister Chromatids (SC) [19, 22, 25]. In the second “train-track” model [23, 26], sister-forks can instead diffuse independently from each other in the 3D space as replication proceeds. Despite various experimental evidence of the interdependent motion of sister-forks in both bacteria [27, 28] and eukaryotes [19, 22, 25], independently moving sister-forks have been also observed in vivo during bacterial replication [23, 26, 29] and in vitro in Xenopus egg extracts [26].

On a larger scale, early microscopy studies also reported the presence of large Replication Foci (RFi), each containing multiple forks, possibly aggregated through a phase separation mechanism [16–18, 30, 31]. While few observations made in budding yeast have supported this model [20], more recent super-resolution microscopy studies in mammals challenged it [21, 32], revealing that individual RFi can be further resolved into single replicons or forks. In this scenario, the larger RFi previously observed may just arise from limited optical resolution combined with the simultaneous activation of multiple origins within the same 3D chromatin domain, without requiring specific, replication-dependent aggregating forces [21, 32].

Given such complex, multi-scale interplay between replication dynamics and 3D chromosome organization, quantitative approaches based on polymer simulations can be useful in testing different models and reconciling conflicting experimental observations. In the last couple of years, a few modeling studies have incorporated explicit replication into coarse-grained polymer frameworks to address these questions in bacteria [33–36] and eukaryotes [25, 31, 37]. In bacteria, where replication starts from a single origin [33–36], these studies suggest that the 3D genome architecture, driven by nucleoid confinement and SMC activity, coupled to replication, leads to the reliable segregation of SCs while promoting some degree of sister-fork colocalization. In eukaryotes, previous models [25, 31, 37] explored idealized—toy—situations of eukaryotic-like replication with several replisomes working in parallel. In particular, our previous work focused on explicitly simulating polymers undergoing self-replication, in analogy with the process of DNA replication in 3D. Employing such a formalism, we demonstrated how the synthesis of two sister chromatids inside a replication bubble can mediate significant local rearrangements of chromatin in S-phase. Interestingly, when introducing multiple origins, we also observed a significant impact on the 3D structure and dynamics at larger scales [25]. However, to systematically characterize such processes, only deterministic and synchronous firing of equally spaced origins was considered. While such results can provide a theoretical basis for how replication reshapes the genome in 3D, it is still unclear how these emerging structures are affected when the stochasticity of the replication timing is introduced. Forte et al*.* [31] characterize instead the energetic requirements to sustain sister-forks association via diffusing particles and their potential dynamics in the assembly of higher-order RFi-like structures. More recently, a new modeling study also address the potential role of DNA replication and forks speed at the domain level [37]. In summary, all of these approaches lack a realistic description of chromosome organization and origin firing that prevents quantitative analysis and comparison with experimental data.

To fill this gap, we develop a genome-wide 3D modeling of replicating chromosomes [25] of the budding yeast *Saccharomyces cerevisiae* for which the spatial architecture and replication are of minor complexity and have been extensively characterized separately. At the nuclear level, the *Saccharomyces cerevisiae* genome exhibits a polymer brush-like, Rabl organization, where all 16 centromeres are attached to the spindle pole body (SPB) via the mitotic spindle [38] while the telomeres are anchored at the nuclear envelope (NE) [39, 40]. Opposite to the SPB lies the nucleolus, a defined subnuclear structure that isolates the ribosomal DNA repeats of chromosome 12 from the rest of the genome [38, 41]. Entering S-phase, the yeast genome is duplicated starting from specific consensus sequences called autonomous replicating sequences (ARS) [42, 43] and whose efficiencies are highly heterogeneous, leading to complex replication patterns [1, 44–48]. While G1 chromosomes lack structural features such as TADs or loops [3], cohesin is gradually loaded on chromatin during S-phase to mediate the cohesion between sister chromatids [49–55] and mitotic condensation via loop extrusion [56–61]. To realistically simulate such a system, we couple a precise description of the hierarchical firing of yeast origins [62, 63] with a coarse-grained polymer model explicitly accounting for chain duplication [25] and for the budding yeast Rabl-like organization [64–68]. This integrated approach enables us to examine the 3D features of *Saccharomyces cerevisiae* during S-phase across multiple scales. Taking advantage of the realistic 1D replication dynamics and genome-wide statistics, we first explore how the two proposed models of sister-fork association impact the 3D chromosome structure at the replicon scale. Furthermore, we compare our predictions with new in vivo Hi-C data from S-phase, which reveal distinct fountain-like patterns around early origins. These patterns are consistent with at least a fraction of sister-forks extruding the newly synthesized SCs during their progression.

Beyond the replicon scale, our framework allows to explore how the physical constraints imposed by the Rabl architecture shape the 3D distribution of forks across the nucleus, potentially affecting the detection of larger RFi containing multiple pairs of sister-forks. Finally, we explore the dynamics of replicating chromosomes, providing quantitative insights on the potential slowdown in the diffusion of chromatin in the presence of active forks and intertwined structures.

## Results

### Modeling replication in space and time

#### A minimal polymer model recapitulates the yeast nuclear architecture in G1

In order to address the role of replication in driving the 3D Genome in S-phase, we first implement a minimal, quantitative genome-wide polymer model of the nuclear architecture of haploid yeast in G1. It aims at capturing the most fundamental structural properties of chromosome organization to serve as a “null” model.

**Fig. 1:**
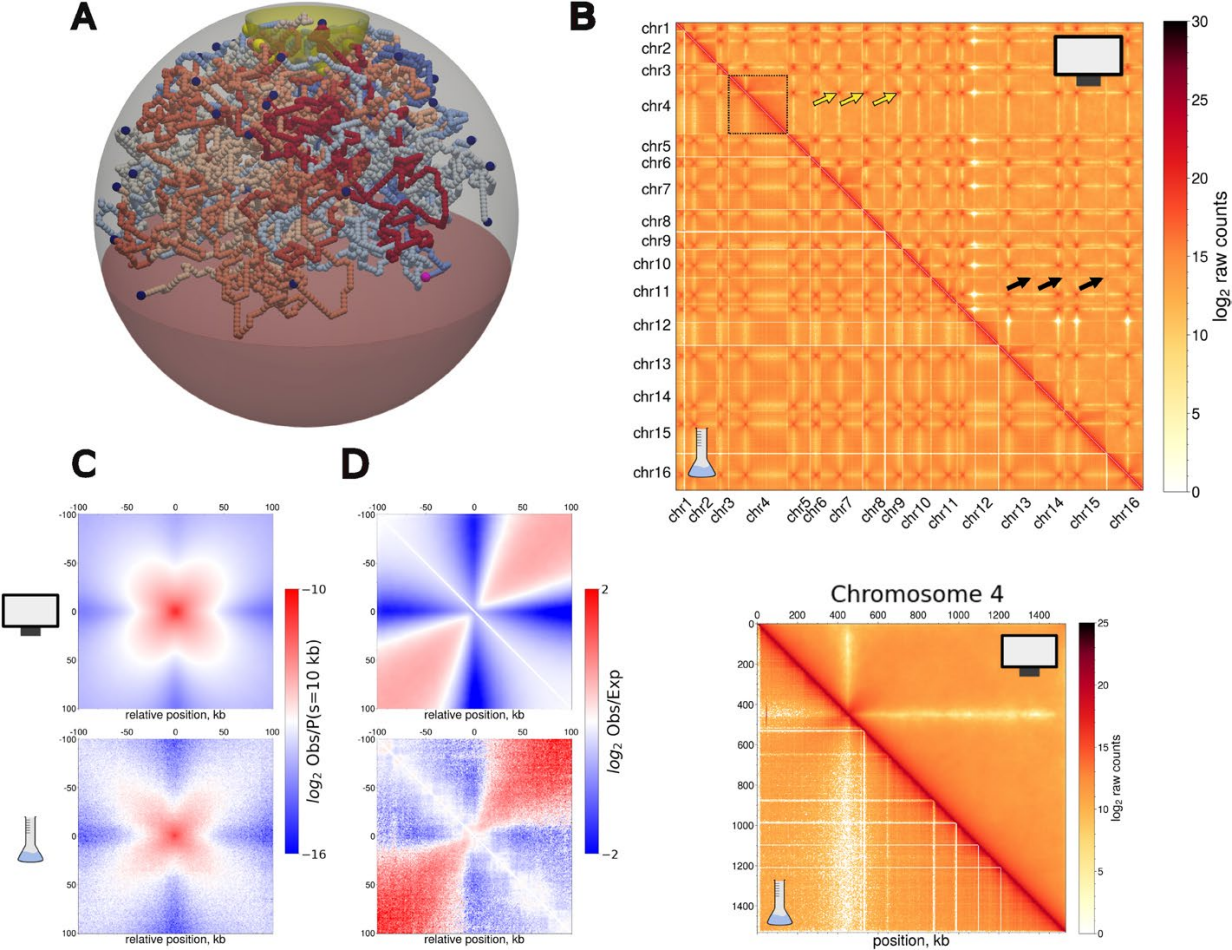
Genome-wide modeling of *Saccharomyces cerevisae* haploid genome in G1. **A** Snapshot of G1-like simulations. Each color indicates a different polymer chain. Centromeres, telomeres and rDNA boundaries are highlighted with larger beads in yellow, dark blue and pink respectively. The red region within the sphere indicates the nucleolus and is inaccessible to other monomers. **B** Top: simulated (upper triangle) and in vivo (lower triangle) Hi-C maps (raw contacts) of the whole genome at a resolution of 16 kb for *r*_*c*_ = 80 nm. Yellow and black arrows indicate inter-centromeres and inter-telomeres contacts respectively. The dashed square on the diagonal highlights chromosome 4, plotted in the bottom panel at 1 kb resolution. **C** Simulated (Top) and experimental (Bottom) off-diagonal inter-chromosomal aggregate plots between all centromeres pairs. The signal is normalized by the *P*(*s* = 10* kb*). **D** Simulated (Top) and experimental (Bottom) on-diagonal aggregate plots (Observed/Expected) around yeast’s 16 centromeres. The number of biological replicates of experimental Hi-C data shown merged in (**B**-**D**) is n = 2

Briefly (see Methods for details), similarly to previous studies [64–68], we model the full *Saccharomyces cerevisae* haploid genome by confining the 16 chromosomes in a spherical volume to mimic the NE (Fig. 1A, Additional file 1: Fig. S1A,B,C). Each chromosome is modeled as a semi-flexible, self-avoiding chain, each monomer encompassing 1 kb and being of size 20 nm. Specific features of the typical yeast Rabl organization [38, 41] such as the clustering of centromeres at SPB and the tethering of telomeres at NE are recovered by adding physical constraints on the corresponding specific regions of each chromosome (Fig. 1A and Additional file 1: Fig. S1B for a more detailed scheme). Finally, we include a minimal description of the nucleolus, that encompasses the Mbp-long rDNA region present on chromosome 12, by including a barrier (“nucleolus wall”) within the nuclear sphere to mimic the 3D space inaccessible to the rest of DNA and by splitting chromosome 12 into two separate chains, each including an rDNA boundary which is tethered to the nucleolus wall (Fig. 1A, Additional file 1: Fig. S1C).

As highlighted previously [66, 67, 69, 70], such a minimal model is able to quantitatively capture the typical polymerbrush-like architecture observed experimentally. In particular, we obtain a Pearson correlation between simulated and experimental full-genome Hi-C maps at 16 kb resolution of 0*.*93 (Fig. 1B Top). Moreover, on this scale, we observe a high frequency of contacts between centromeres (Fig. 1B, yellow arrows). This predicted enrichment in contacts arises from the imposed colocalization of centromeres around the SPB and resembles the experimental observations (Fig. 1C). Similarly, the focal telomere-telomere contacts observed in Hi-C are captured (Fig. 1B, black arrows) just by imposing their confinement at NE without adding any attractive energy.

In addition to the overall nuclear organization, our model also quantitatively reproduces the intra-chromosome architectures in G1 (Fig. 1B Bottom, Additional file 1: Fig. S1D and S2) with an average Pearson correlation for Hi-C maps at the single chromosome level of 0*.*88 (see Methods and Additional file 2: Table S1 for more details). Due to the imposed Rabl-like architecture, the model predictions match the average enrichment of contacts between the arms of the same chromosome around centromeres (Fig. 1D) observed experimentally. Moreover, our *P*(*s*) curves correctly capture enrichment at longer distances due to telomere attachment at NE (Additional file 1: Fig. S2C).

In summary, our minimal “null” G1 model captures the main features of budding yeast 3D nuclear architecture, allowing us to investigate its interplay with explicit chromosome replication.

#### Modeling replication dynamics and explicit chain duplication during S-phase

**Fig. 2:**
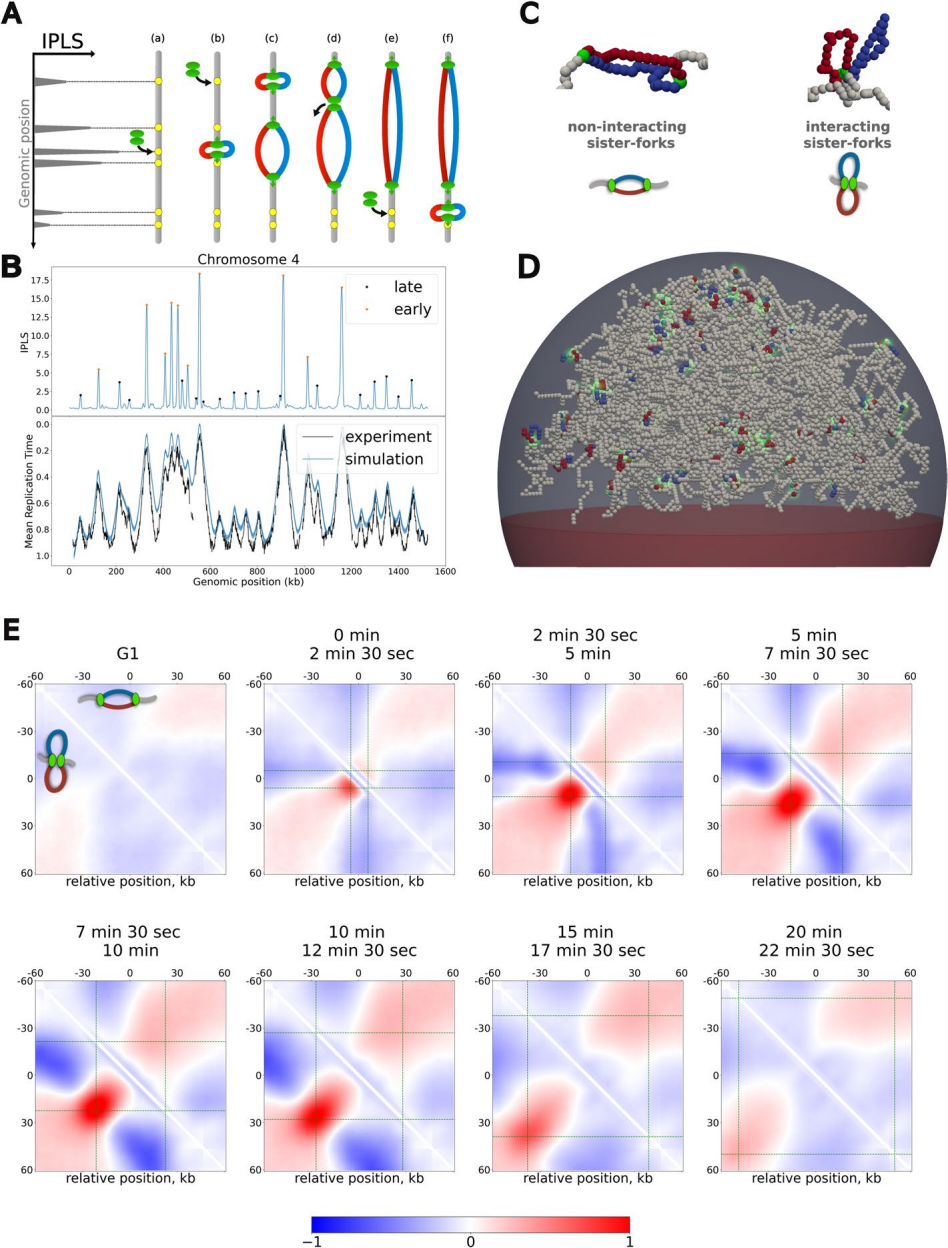
Spatio-temporal whole-genome model of yeast DNA replication. **A** Scheme of the 1D replication model by Arbona et al*.* [62, 63]. Origins are drawn with replacement from the IPLS signal (Left): (a, b) they “entrap” one firing factor leading to the opening of a replication bubble with two diverging forks; (c) Replication bubbles grow as forks progress, and new firing events might be hindered by the limited number of diffusing factors; (d) Converging bubbles (merging events) release a diffusing factor that can be employed to fire a new unreplicated origin (e, f). **B** IPLS of chromosome 4 (Top) and corresponding predictions of the mean replication time (Bottom, blue line) compared with the experimental MRT (from [71]) (Bottom, black line). Orange and black circles over *IPLS* peaks indicate origin classified as early and late respectively (see Methods). **C** The two investigated scenarios for the local sister-forks spatial organization with illustrative snapshots from simulations and corresponding scheme. Red and blue monomers correspond to the two replicated sister chromatids, light-gray to the unreplicated monomers. **D** Snapshot of a full genome simulation with chromosomes undergoing replication in the interacting sister-forks scenario. Green halos indicate replication forks location. **E** Average normalized (log_2_ Observed over Expected) contact maps around early replicating origins in the non-interacting (upper triangle) and interacting (lower triangle) scenarios at different times after the beginning of S-phase. Each map was computed aggregating frames within time windows indicated above (2 min and 30 s. each, see Methods). G1 corresponds to the unreplicated G1-like configurations. Green dashed lines indicate the expected average forks positioning at the corresponding time interval

To model the yeast 3D genome during S-phase, we include explicit polymer duplication to simulate the progressive synthesis of two sister chromatids (SCs) from each “maternal”—G1—chromosome (Fig. 2A). This is achieved by employing a specialized class of polymers that self-replicate from predefined genomic positions, the origins of replication [25]. Locally, origin firing consists of adding a new monomer and connecting it to the maternal chain, leading to the formation of a replication bubble (Fig. 2C, D, see Methods for details). The monomers connected at the extremities of this bubble are the replication forks (green monomers in Fig. 2C). Further duplication of such triple connectivity accounts for fork progression along the genome which we assume to be stochastic and happening at constant average speed (*v* = 2*.*2 kb/min [44]). In such a formalism, multiple growing bubbles can be easily introduced and tracked, with converging bubbles being merged into larger loops (Fig. 2A). To simplify, we maintain the volume of the confining sphere identical all long the simulation while in vivo the nuclear size increases during replication [72].

In one simulated trajectory, the dynamics of chain duplication thus depends on the set of origins that will be fired and their timing of firing. To account quantitatively for the correct firing dynamics in yeast, we couple our 3D framework with state-of-the-art modeling of 1D replication dynamics along the genome. Briefly, we integrate the stochastic model of eukaryotic DNA replication by Arbona et al*.* [62, 63] (Fig. 2A) where the firing is modeled as a bimolecular reaction between a set of putative origins and a limiting number of firing factors (see Methods for details). The positions of potential origins (p-ori) are randomly selected among all monomers with a probability proportional to the so-called “Initiation Probability Landscape Signal” (*IPLS*) (Fig. 2A, Left and B, Top) which is inferred from replication data using a neural network [63]. The peaks in *IPLS* correspond to origins of replication and mostly coincide with the yeast autonomously replicating sequences (ARS) (Additional file 1: Fig. S3) from the Oridb database [42].

In particular, using *IPLS* (Fig. 2B Top) and parameters inferred by Arbona et al*.* in [63], our model of replication dynamics well reproduces the MRT profiles observed in yeast throughout the genome (Fig. 2B Bottom, Additional file 1: Fig. S4) and predicts an S-phase of duration ∼ 30 min (Additional file 1: Fig. S5) typical of the yeast cell cycle [73]. Over the 726 inferred origins, we define sets of early and late origins (Fig. 2B and Additional file 1: Fig. S3, see Methods) depending on the peak height in *IPLS*, early origins (111 in total) having a very high probability to be fired at each S-phase (90%), while late origins (615 in total) fired only occasionally (60%).

Equipped with a modeling framework with realistic 1D replication dynamics and 3D chromatin folding within the nucleus, we next investigate how replication and chain duplication may impact genome organization during S-phase at different scales. In particular, we consider two scenarios (Fig. 2C): a first one where sister-forks evolve independently of each other (non-interacting scenario) and a second one where they are enforced to remain spatially colocalized (interacting scenario) until their replication bubble dissociates or merges with another bubble (see Methods). For each scenario, we simulate 300 S-phase trajectories (Additional file 2: Table S2), each initialized with a random G1-like configuration, and we track how the 3D chromosome organization evolves as a function of time after starting the replication process (Fig. 2D, Additional file 3: Video S1, Additional file 4: Video S2).

### Replication impacts transiently the local chromatin organization around origins

We first focus on the local structural impacts of the formation of replication bubbles with or without interactions between sister-forks due to forks passage. From our simulations, we compute balanced Hi-C-like maps (see Methods) at different time points along the S-phase (Additional file 1: Fig. S6).

#### In silico* prediction of chromatin fountains around early replicating origins*

As early origins are fired frequently and more synchronously, we expect the surrounding genomic regions to be more exposed to any structural effects that fork passages may induce. In Fig. 2E, we plot the time evolution of the average contact enrichment around early origins in the interacting and non-interacting forks scenarios. We observe the emergence of typical, replication-dependent patterns that form very early in S-phase, evolve dynamically and finally progressively disappear as replication ends. Remarkably, both scenarios lead to qualitatively different features.

In the non-interacting case (upper triangles in Fig. 2E), we detect a homogeneous enrichment of contacts in a band perpendicular to the main diagonal and centered around the origin whose width is related to the average replicon size at a given time around the origin (green dashed lines in Fig. 2E). This mild increase is related to the formation of replication bubbles around early origins [25]: the resulting ring-like topology of the polymer leads to an effective, entropically-driven compaction of the replicon compared to the rest of the unreplicated, linear polymer chain.

In the interacting scenario (lower triangles in Fig. 2E), a stronger and more localized enrichment is clearly visible. This enrichment is maximal between the average positions of the replication forks and progresses perpendicular to the main diagonal as replication proceeds. This typical signature of a fountain-like pattern, as also observed around cohesin loading sites [74–77], results from the 3D colocalization of the two sister forks combined with their symmetric progression throughout the genome around the origin with time. This leads to an effective loop extrusion mechanism [22, 25].

#### Replication-dependent and cohesin-independent chromatin fountains are observed in vivo around early origins

We then investigate whether the predicted patterns can be detected in in vivo Hi-C data during S-phase. Although the presence of mild fountain-like patterns around origins in yeast was already reported in our previous study [25] using publicly available data in WT cells [52, 59, 61], it was not possible to directly establish a causal-effect relationship between these observations on Hi-C maps and the replication process, as well as to rule out possible confounding effects such as the concomitant presence of extruding cohesins.

For this reason, we generate new Hi-C datasets during early S-phase in WT and mutant yeast strains to isolate the contribution of replication and decouple the emerging signal from concurrent biological processes (see Methods, Additional file 1: Fig. S7 and S8). For this, cells were arrested in G1 using alpha-factor and then released in S-phase for 20 min before fixation.

##### Enrichment around early origins in vivo is replication-dependent

**Fig. 3:**
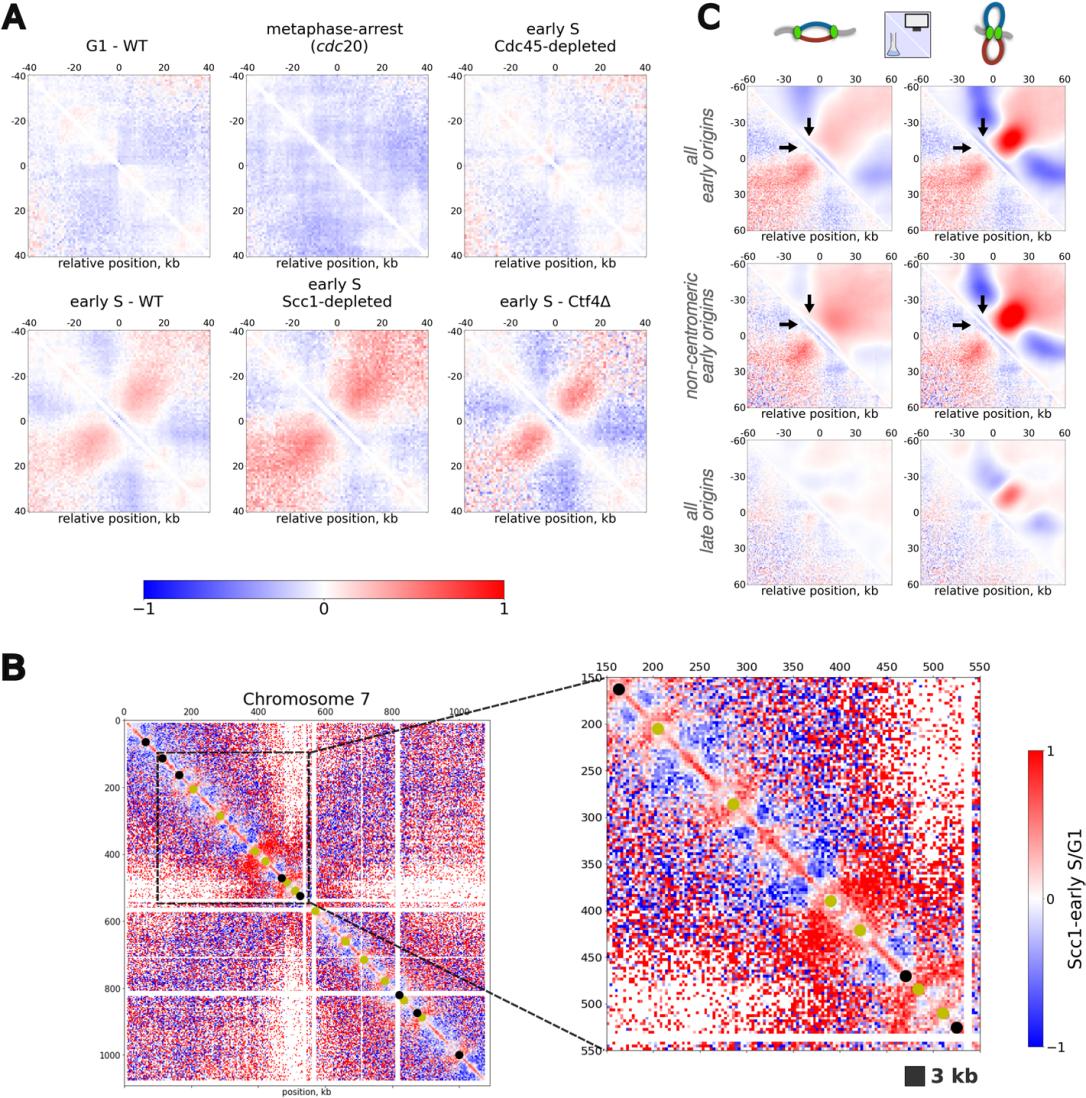
Comparison with in vivo data and forks spatial distribution. **A** Average normalized (log_2_ Observed over Expected) contact maps around early replicating origins for in vivo Hi-C data for different conditions. **B** log_2_ ratio between Scc1-depleted and G1 Hi-C maps for chromosome 7. In this case, maps were plotted at 3 kb resolution showing the full chromosome (Left) and a 400 kb region (Right). Yellow and black dots indicate early and late replicating origins respectively. **C** Comparison between experimental aggregates of Scc1-depleted cells and simulation using the best matching time-interval from Fig. 2F (from *t* = 5 min to *t* = 7 min and 30, see Additional file 1: Fig. S9A). Aggregates were computed around all early, only non-centromeric (centromeres at a distance > 50 kb) and all late origins for the two scenarios of sister-forks organization. Black arrows highlight the qualitative differences between the two simulated signals with a depletion and enrichment at the loop basis in the interacting and non-interacting scenario respectively. The numbers of biological replicates of experimental Hi-C data shown merged in (**A**-**C**) are: G1-WT n = 2, early S-WT n = 3, early S Cdc45-depleted n = 2, early S Scc1-depleted n = 1, early S-Ctf4∆ n = 1, metaphase-arrested (*cdc20*) n = 2

In WT condition, analysis of the Hi-C data in early S-phase, shows the presence of a fountain-like pattern around early origins (Fig. 3A Bottom Left) with an enrichment around 10-30 kb. This structure appears specifically during S-phase as it is not visible in G1 (Fig. 3A Top Left) or G2/M-arrested (*cdc20*, Fig. 3A Top center) cells. Remarkably, the observed signal at this early-S time-point appears significantly stronger than our previous report [25] using publicly-available Micro-C data [61].

To investigate the role of replication in the formation of the fountains, we perform similar experiments but in Cdc45-depleted cells using an auxin-degron system. Cdc45 being a component of the replicative helicase CMG, it is essential for replication bubble opening at origins [78]. Cdc45-depleted cells thus progress towards G2/M but without performing DNA replication and in the absence of SC cohesion. In particular, it was observed that, in this case, chromosomes still engage in a mitotic-like compaction via cohesin-mediated loop extrusion activity [56, 58].

Measuring the Hi-C maps, as in the WT, 20 min after release from G1 and depletion in Cdc45 (Additional file 1: Fig. S7C), we observe complete loss of the fountain signal around early origins (Fig. 3A Top Right) after Cdc45 depletion.

Overall, this result demonstrates that the progression along the cell cycle (Cdc45-depleted) nor the presence of a second SC (*cdc20*) are sufficient to retain the fountain signal, which requires ongoing replication and is observed only transiently during S-phase.

##### Enrichment around early origins in vivo is cohesin-independent

Despite such findings, it is still unclear whether the emerging pattern is a direct consequence of the 3D organization of sister-forks, or driven by other concurring processes also happening during S-phase. In particular, in budding yeast, cohesin-mediated SCs cohesion and intrachromatid loop-extrusion constitute two well-established processes that impact the 3D chromosome organization and that initiate during S-phase [49–55]. In particular, cohesins start extruding during early S leading to the formation of loops between specific genomic regions called cohesin associated regions (CARs) [61] (Additional file 1: Fig. S9B).

The early-S Cdc45-depleted and metaphase-arrested maps presented in Fig. 3A demonstrate that the fountains observed in WT early-S are not just a simple by-product of some trivial correlations between CARs and early origins positioning along the genome, as a complete loss of fountains occurred in these cases while chromatin loops at CARs are still preserved (Additional file 1: Fig. S9B). However, this does not exclude that some cohesins might also be specifically recruited at replication forks and contribute to the signal.

To explore this hypothesis, we analyze early-S phase Hi-C map from cells partially depleted of Scc1, a cohesin subunit, using an auxin-degron system (Additional file 1: Fig. S7D). We first verify that such a depletion reduces strongly the cohesin loop extruding activity and removes the loops between CARs in the Hi-C map, while their signature remains present in WT, Cdc45-depleted cells (Additional file 1: Fig. S9B). Around early origins on the contrary, fountains are persistent to cohesin depletion (Fig. 3A Bottom Middle). Unexpectedly, the average fountain signal around origins appears enhanced in the absence of cohesin compared to WT. We speculate that the average decrease in contact frequency (*P*(*s*) curve) due to the depletion of loops on chromatin, may play a role when normalizing the maps by the expected value (see Methods). As a result, replication-driven fountains are likely enhanced due to less interplay with loops occurring at similar genomic scale (10s of kb). Interestingly, due to better contrast, fountains can be visualized not only at the population level, but also at the single origin level when we normalize the Hi-C map for Scc1-depleted cells in early-S by the one in G1. In this case, most early origins exhibit a fountain-like pattern (Fig. 3B, Additional file 1: Fig. S10), that are absent when repeating the analysis using metaphase-arrested cells (*cdc20*, Additional file 1: Fig. S11).

Overall, this suggests that the fountain signal does not depend on cohesin loading and loop extrusion activity.

##### Enrichment around early origins in vivo is Ctf4-independent

Given experimental evidence of its ability to oligomerize in vitro [79], Ctf4, a component of the replisome, has been proposed as a possible mediator of sister-fork interactions. We thus perform Hi-C experiments as before but in Ctf4-KO cells that are still progressing through S-phase (Additional file 1: Fig. S7E). In this case, we still observe loops around CARs (Additional file 1: Fig. S9B) and more importantly, the characteristic enrichment around early origins is still present (Fig. 3A, Bottom Left), suggesting that Ctf4 might not be a driver of the fountain pattern. Interestingly, Ctf4 depletion leads on average to smaller replicon sizes (Additional file 1: Fig. S12). We hypothesize that a decrease in the fork progression speed may thus explain the differences in size and strength between fountains in WT and Ctf4-KO.

#### Comparisons with simulations suggest that sister-forks can interact *in vivo*

The Scc1-depleted maps discussed above offer the best comparison to our polymer model, which does not account for cohesin-mediated processes such as cohesion or loop-extrusion and only implements the formation and growth of replication bubbles.

To make the most meaningful comparison possible, we first have to infer the effective time in our simulations corresponding to the early-S stage in the experiments where cells do not start replication immediately after release. To do so, we estimate and compare the simulated and experimental average number of Hi-C reads around early origins, which reflects the local probability of having been replicated (see Additional file 1: Fig. S9A for a more detailed description of the method used). We find a corresponding time interval in our simulations between 5 and 7.5 min (Fig. 2E, top Right).

##### Around early replicating origins

We then compare the contact signals observed around early origins (Fig. 3C). Overall (Top panels), experimental data are qualitatively more similar to the fountains observed in the interacting fork scenario with common characteristic features: (1) a well-localized enrichment perpendicular to the main diagonal, corresponding to an average replicon size of 30 kb, and (2) a depletion of the signal at the loop basis (see black arrows in Fig. 3C Top).

Since early origins are more densely located around centromeres, to avoid any possible structural artifacts related to the contact enrichment observed around centromeres (Fig. 1D), we also compare specifically the signal for noncentromeric, early origins (Fig. 3C Middle). They are characterized on average by greater inter-origin distances and thus are likely to have experienced fewer merging events between nearby replicons in early-S. In vivo, we still observed a fountain-like pattern which also qualitatively resembles the predictions of the interacting fork scenario.

Overall, the interacting fork predictions are thus more compatible with the fountain shape observed in the experimental data.

However, the strength of the enrichment signal is much stronger in the predictions than in the experiments (see also Additional file 1: Fig. S9, S13). This may suggest an intermediate scenario where not all the sister-forks are paired. Indeed, our working hypothesis of strictly-bound sister forks is likely to be a strong approximation of the real biological system, as it has been experimentally observed that replication with independently moving forks is still viable [26].

We address this hypothesis by mixing predicted contact maps of the interacting and non-interacting fork cases at different ratios (Additional file 1: Fig. S9F). Interestingly, a small percentage of interacting forks signal (≥ 20%) is already sufficient to retain the localized enrichment perpendicular to the main diagonal (fountain pattern) while lowering the absolute value of the enrichment.

We also explore alternative confounding factors that may affect the predicted fountain strengths. While our model captures the experimental 1D dynamics and hierarchical firing of origins, all of the simulated trajectories are perfectly synchronized and enter replication at the same time. This is likely not the case the experiments in which the beginning of the S-phase in cycling cells or in cells released from G1 after synchronization is likely to be more heterogeneous [57, 61]. If we mix predictions for early-S with some for G1 (Additional file 1: Fig. S9C), we also observe a reduction of the signal around early origins. Interestingly, the fountain pattern is very robust even at strong dilution, suggesting that such replication-dependent signals may also be captured in a non-ideally synchronized system.

Finally, we investigate the impact of the model parameters used in estimating the in silico contact maps: the radius of capture (Additional file 1: Fig. S9E) which is the maximal distance between two monomers used to define a contact; and the length of the time intervals (Additional file 1: Fig. S9D) used to compute maps. While these parameters have a quantitative impact on the enrichment, they both maintain the overall qualitative difference between the two fork scenarios.

Overall, experimental data around early origins are consistent with the presence of a significant population of interacting sister-forks in vivo.

##### Around late replicating origins

We then investigate if our model also well captures the signal around late replicating origins (Fig. 2B, Top). In the in vivo Scc1-depleted maps, we record only a faint signal around late origins, both in terms of size and strength (Fig. 3C Bottom, lower triangle). Predictions in both sister-fork scenarios show strong differences. In the interacting fork case, we continue to record a mild fountain-like pattern that is not present in the experiment (Fig. 3B Bottom Right). On the other hand, in the non-interacting case (Fig. 3C Bottom Left), only a faint signal is present, resembling more the experimental observation. Mixed scenarios with a majority of non-interacting cells are also consistent with the experiments (Additional file 1: Fig. S14). This is consistent with what is observed locally at late origins in the differential plots (Scc1-depleted over G1) of Fig. 3B and Additional file 1: Fig. S10 (black points). While we do observe enrichment around some of the late origins, in most cases no specific pattern is detected. Overall, this suggests that interactions between sister-forks is less likely when fired from a late origin.

### The spatial organization of replication forks inside the nucleus is dynamic and heterogeneous

Thanks to our genome-wide modeling of the Rabl-like yeast genome organization, we can then ask where exactly replication occurs in the 3D nuclear space along the S-phase.

#### A replication “wave” occurs across the nucleus

**Fig. 4:**
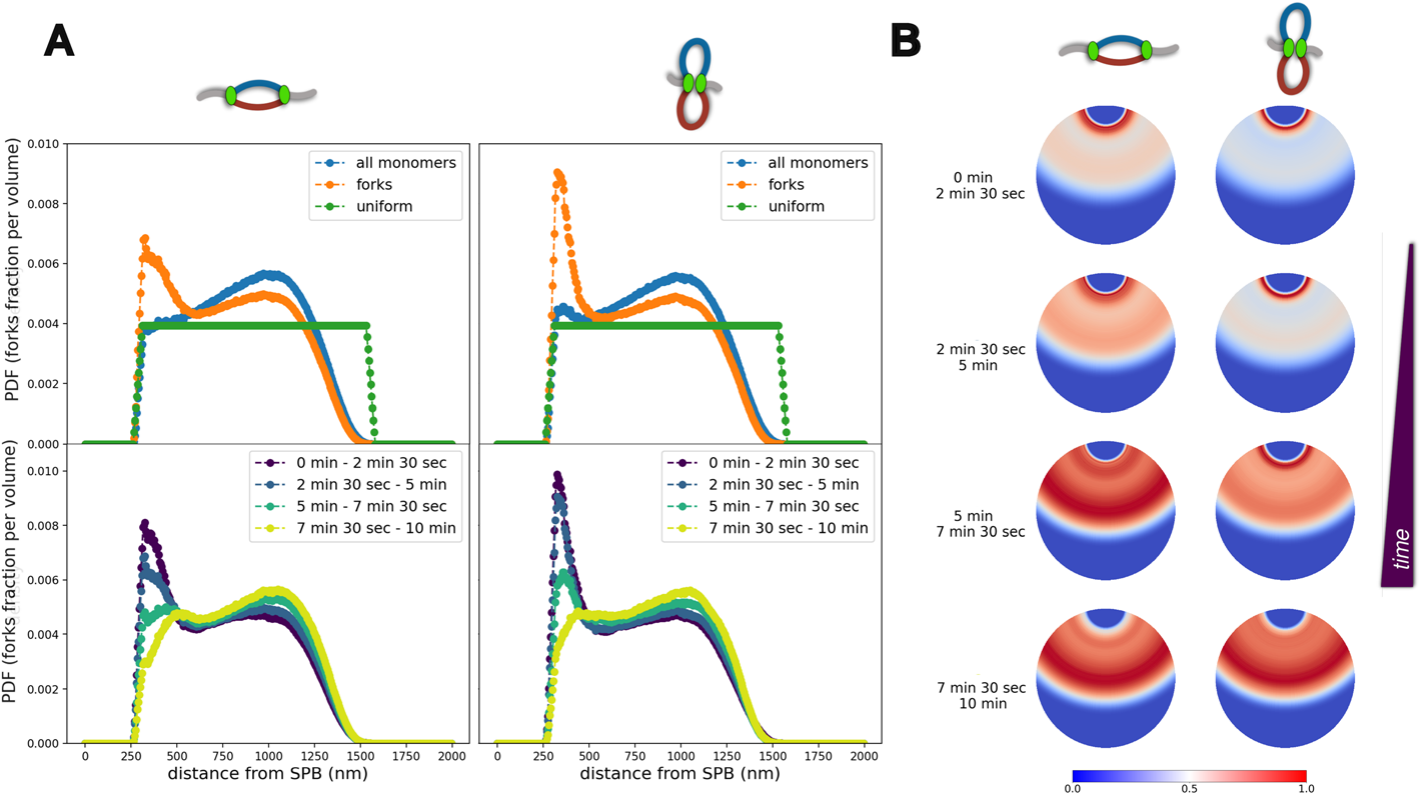
Forks spatial distribution. **A** Top: Probability Distribution Function (PDF) for a monomer to be at a distance *r* from the SPB: for all the monomers (blue) or only forks (orange), and compared with a uniform distribution (green). The computation includes the simulation frames between *t* = 2 min and 30 s to *t* = 5 min in the non-interacting (Left) and interacting (Right) sister-forks scenarios. Bottom: Same as (**A**) for replication forks at different time intervals. **B** 2D graphical representation of the time evolution of forks densities plotted in the Bottom panels of (**A**). Note that the color scale used to plot the density profile of each time interval is normalized between 0 and 1

We start by investigating the location of replication forks inside the nucleus. In particular, we compute the nuclear density of forks and monomers as a function of the 3D distance from the SPB for both interacting fork scenarios (Fig. 4, Additional file 1: Fig. S15, Additional file 5: Video S3, Additional file 6: Video S4).

We observe that the spatial distribution of all the monomers is heterogeneous within the nucleus as a result of the Rabl organization, with an enrichment around the equatorial plane (Fig. 4A, Top, blue lines). Interestingly, this overall density evolves during S-phase, particularly in the interacting fork case with a slight enrichment close to the SPB in early-S that gradually vanishes at longer times (Additional file 1: Fig. S15E), an effect that is not present for non-interacting sister forks (Additional file 1: Fig. S15D,E). This demonstrates that replication not only impacts locally the organization around origins (see above), but leads to a global rearrangement of chromatin within the nucleus.

When we focus on fork positioning, we observe a strong enrichment in early S around SPB (Fig. 4A Top, orange curves). This is consistent with the higher abundance of early firing origins close to the centromeres. Interestingly, such an effect is stronger in the interacting forks scenario. Such an enhanced localization around centromeres is gradually lost as replication progresses, with a relocation toward the equatorial plane (Fig. 4A Bottom and B and Additional file 1: Fig. S15). Indeed, at larger times, centromeric regions are likely to be replicated and forks tend to propagate more in the direction of telomeres. Moreover, new firing events of non-centromeric regions may further increase this effect.

Our results demonstrate the presence of a replication “wave” during S-phase in yeast, as already suggested by others on the basis of MRT and Hi-C data [57]. More precisely, our simulations indicate that, on average, starting from an enrichment of replication forks at one pole (the SPB) in early S-phase, forks are redistributed in mid S-phase within the nucleus with a distribution comparable to the overall average chromatin distribution (blue curves in Fig. 4A, Top). In late S-phase (Fig. Additional file 1: Fig. S15A,B), when the number of forks is low (Additional file 1: Fig. S5), we observe a preferential enrichment at larger distances from the SPB. Interestingly, our results imply that such non-trivial re-distribution of forks naturally emerge when combining G1 nuclear architecture with the correct replication timing for each duplicating chromosome. Such a “wave” can also be partially observed at the level of an individual trajectory (Additional file 5: Video S3, Additional file 6: Video S4). However, we predict that fluctuations due to the stochastic replication dynamics and 3D diffusion of monomers may strongly impact the ability to visualize such a redistribution in vivo at the single-cell level.

#### Replication forks may form small foci independently of specific aggregating forces

In the previous section, we observed a complex and heterogeneous distribution of replication forks within the nucleus driven in part by the Rabl architecture. Here, we investigate whether this peculiar spatial organization can explain by itself the presence of replication foci (RFi), where forks colocalize, as observed experimentally [20].

##### Characterization of replication foci in silico

**Fig. 5:**
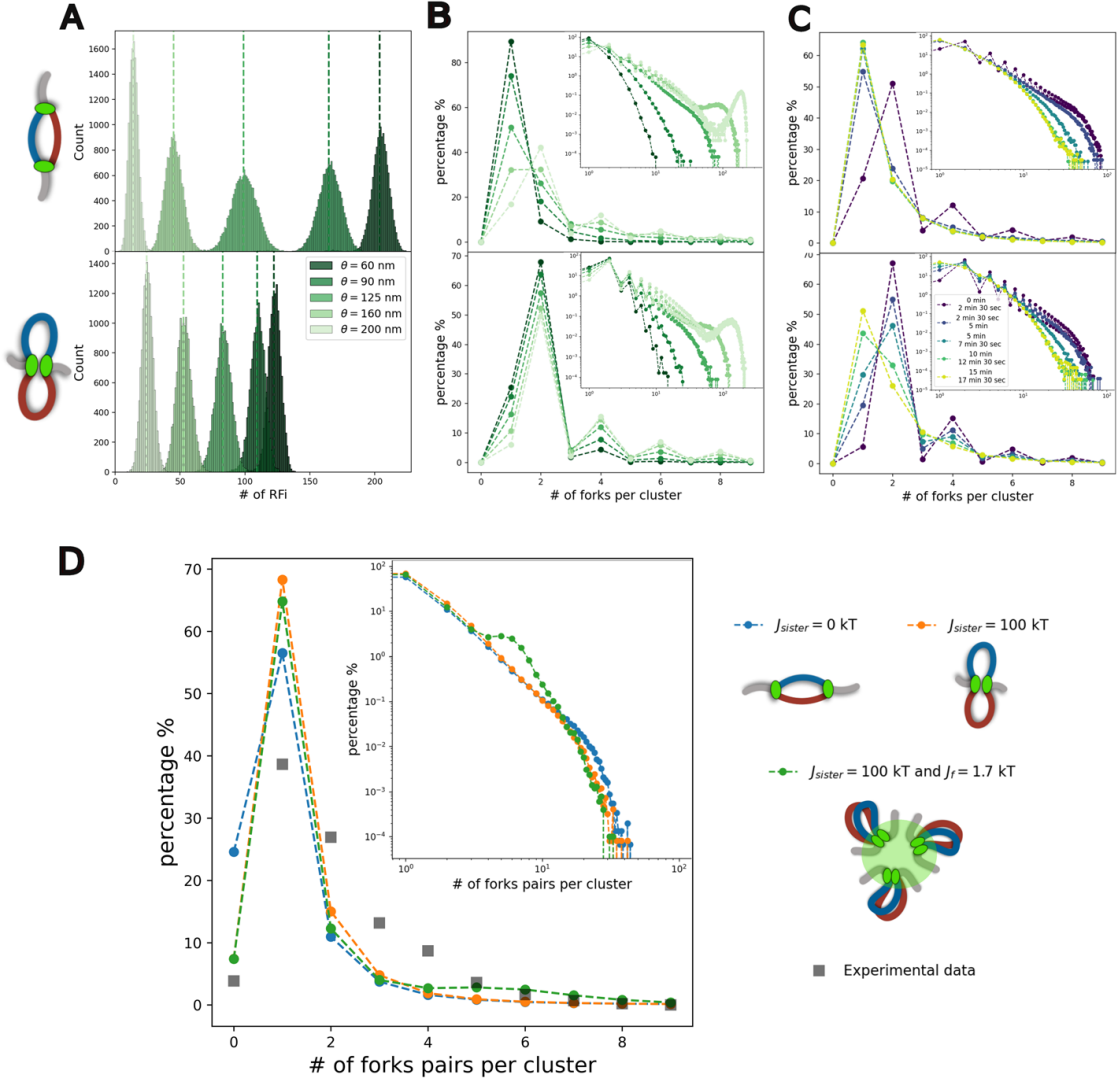
Detection of RFi within the nucleus during S-phase. **A** Distributions of the number of distinct RFi detected at a given resolution *θ* in early-S simulations (between *t* = 2 min and 30 s and *t* = 3 min and 45 s) for non-interacting (Top) and interacting (Bottom) sister-forks. The dashed lines correspond to the mean value of each corresponding distribution. **B** Percentage of clusters containing a given number of forks for simulations in early-S (between *t* = 2 min and 30 s and *t* = 3 min and 45 s) for non-interacting (Top) and interacting (Bottom) sister-forks and for various resolutions *θ*. The inside panels show the distributions in logarithmic scale, including the probability for higher number of forks per cluster. **C** Percentage of clusters containing a given number of forks for different time windows in the non-interacting (Top) and interacting (Bottom) sister-fork cases. The inside panels show the distributions in logarithmic scale. **D** Left: percentage of clusters containing a given number of forks pairs. Gray squares indicate the experimental estimates by Saner et al*.* (see Methods). Simulated data in early-S (between *t* = 2 min and 30 s and *t* = 3 min and 45 s) was analyzed to strictly obtain even number of forks per cluster (see Methods). Right: schemes and color relative to the three models explored in panel D

To define and detect RFi in each simulation snapshot, we consider the independent components of the corresponding adjacency matrix *A*_*i,j*_, where *A*_*i,j*_ = 1 if forks *i* and *j* are distant by less than a threshold distance *θ* (*A*_*i,j*_ = 0 otherwise) (see Methods). *θ* may represent the typical spatial resolution of any experiments that allow to “visualize” the replication fork. For example, for fluorescence-based microscopy, we might expect *θ* to vary from 60 nm (super-resolution-like) to 200 nm (confocal-resolution-like). For both scenarios of sister-forks association, we computed the distribution of RFi during early S-phase, considering all frames between 2*.*5 and 5 min (Fig. 5A,B) for several *θ* values.

In the non-interacting scenario (Fig. 5A, Top), at high resolution (*θ* = 60 nm), most of the individual forks can be resolved. Consequently, the average total number of detected RFi in the system is approximately equal to the average total number of replicating forks at that corresponding moment of S-phase. In the interacting sister-fork case (Fig. 5A, Bottom), as expected, at *θ* = 60 nm, the total number of RFis now corresponds mainly to the number of replisomes. As *θ* increases and the resolution is reduced, additional links are incorporated into the adjacency matrix *A*_*i,j*_, leading to a progressive shift towards lower values for the distribution of the number of RFi (Fig. 5A). Interestingly, for low *θ* values (≤ 160 nm), the interacting scenario always results in fewer detected RFi, each containing more forks on average due to the forced colocalization of sister-forks. However, for low resolution (*θ* ≥ 160 nm), observed RFi in the non-interacting case are less numerous (Fig. 5A) and larger on average. This is likely due to the more homogeneous distribution of forks in the non-interacting scenario (Fig. 4).

To further characterize the cluster population, we compute the probability distribution of the RFi sizes (Fig. 5B) at different resolutions *θ*. In the non-interacting case and at lower resolutions (*θ* < 125 nm), most detected RFi consist of a single diffusing fork (Fig. 5B, Top). Increasing *θ* leads to the detection of some larger clusters. In particular, the probability of detecting sister-forks within the same cluster is enhanced, thus biasing the distribution to even numbers of forks (Fig. 5B, Top, lighter green points). In the interacting forks scenario, such a bias to even number is already present at high resolution (Fig. 5B Bottom, darker green points) and the presence of clusters containing 4, 6 or 8 forks is not rare. Since we do not introduce interactions between the two remaining diverging forks after a termination event (see Methods), even in presence of sister-forks interactions, odd numbers of forks can be detected in our simulations (Fig. 5C Bottom) Interestingly, at very low resolution (*θ* ≥ 160 nm), in both scenarios, very large RFis containing a large fraction of the active forks may be observed (secondary peaks in the inset panels Fig. 5B).

Fixing *θ* = 125 nm to mirror the optical resolution in the experimental work by Saner et al*.* [20] (see below), we analyze how the sizes of RFis change during S-phase. In the non-interacting case, at very early S, the distribution shows again a bias in favor of even values (Fig. 5C Top panels, dark violet points) due to the small average replicon size and the subsequent proximity of sister-forks that are detected colocalized at such a resolution. The high density of forks in centromeric regions (as shown in Fig. 3C,D) also increases the probability of observing clusters (over 40% with 4 forks or more). This is rapidly lost as replication proceeds, leading to the detection of a high percentage of single-forks (> 60%), and to the gradual diminution of large RFis (Fig. 5C top panels). In the presence of sister-fork interactions, the bias toward even cluster sizes persists until 5-7 min (Fig. 5C Bottom panels) after the beginning of S-phase. After, termination events where nearby replication bubbles merge lead to the loss of sister-fork interactions and thus to an increasing number of RFi with single fork (Fig. 5C bottom panels, yellow and green points).

In summary, our in silico predictions indicate that random collisions of diffusing forks or sister-fork pairs may indeed lead to the significant detection of RFi containing several forks or replisomes. Clusters of large sizes are more abundantly detected in early-S when the spatial distribution of forks is enriched around the SPB (Fig. 4).

##### Comparison with experiment

We test whether the predicted, resolution-dependent colocalization of multiple freely diffusing forks is consistent with experimental observations made by Saner et al. [20]. Using super-resolution microscopy (SIM technology) on GFP-tagged PCNA in early S, a key component of replisomes, the authors identified RFi at different time points along S-phase. For one time point where the total number of RFi is maximal, they inferred the number of sister-fork pairs inside a given RFi based on its overall fluorescence intensity (gray squares in Fig. 5D). Note that the inferred distribution is based on an in silico estimation of the total number of forks [80] and—motivated by their previous live-cell imaging observation [19]—on the assumption that sister-forks always co-localize [19] (see Methods). Such a minimal approach does not account that termination events (encounters of convergent forks) can occur even in early S-phase. Aware of such limitations in the experimental estimates, we mirror the data analysis performed by Saner et al*.* by strictly casting the numbers of forks within RFi to even numbers (see Methods).

In Fig. 5D, we estimate the distributions of the number of fork pairs inside our detected RFis (for *θ* = 125 nm, the typical spatial resolution of 3D-SIM [81]) at a time (2-4 min after the start of S-phase simulations) where the total number of replicating forks in silico is maximal (Additional file 1: Fig. S5) and comparable to the estimation made by Saner et al*.* at the time of their measurements (∼ 240 excluding rDNA-related forks). Interestingly, in our simulations, under these conditions, the average number of detected RFis is ∼ 80 (respectively ∼ 100) in the interacting sister-fork scenario (resp. non-interacting scenario), very close to the experimental observation (∼ 75). Both scenarios of sister-forks association exhibit similar trends with a peak at 1 pair per cluster, as observed experimentally, and follow by a rapid decrease for higher numbers of pairs, failing to capture the significant probability of having RFi of intermediate sizes (2-4 pairs) inferred by Saner et al.

We thus wonder if such an underestimation of detected clusters of intermediate sizes in silico may be explained by the presence in vivo of aggregating forces that might stabilize larger clusters. Therefore, in addition to sister-forks interactions, we included non-specific pair-wise contact interactions between all the forks (see Methods, Additional file 1: Fig. S16). At the Hi-C level, we predict an enhancement of contacts between early origins (Additional file 1: Fig. S17) as the strength of non-specific interactions *J*_*f*_ increases, which becomes significant for *J*_*f*_ ≥ 1*.*75 kT. At the level of the distribution of RFi sizes (Additional file 1: Fig. S18), we observe only an enrichment of large clusters (5-15 pairs) for *J*_*f*_ = 1*.*75 kT, and intermediate sizes (3-4 pairs) become more frequent for stronger *J*_*f*_.

Experimental Hi-C data in early S (Additional file 1: Fig. S17D) do not exhibit significant contacts between early origins, suggesting that *J*_*f*_ < 1*.*75 kT. However, at such interaction strength, the predicted frequency of intermediate RFi sizes is not compatible with microscopy data (Fig. 5D, Additional file 1: Fig. S18).

In summary, the colocalization of replicating forks into RFi predicted by our modeling, either just by random collisions or mediated by aggregating non-specific forces, are not compatible with the super-resolution microscopy data of Saner et al*.* (see Discussion and Additional file 1: Fig.S19).

### Replication impacts chromatin mobility

Finally, to complete the spatio-temporal description of replicating chromosomes, we investigate the impact of replication on chromatin mobility.

#### Overall slowing down of chromatin diffusion due to catenations between sister chromatids

**Fig. 6:**
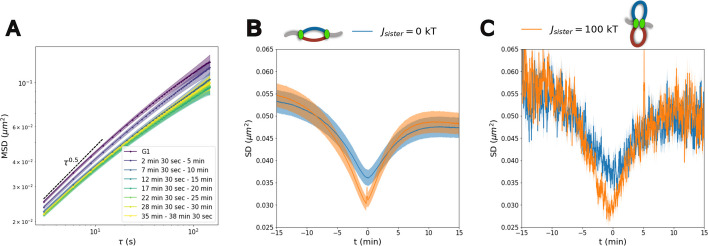
Chromosome dynamics during S-phase. **A** Mean Squared Displacement (*MSD*) for various time windows in S-phase in the case of non-interacting sister forks (*J*_*sister*_ = 0 kT). Curves correspond to an average across all the monomers and all the simulated trajectories. **B** Average Square Displacement *SD*(*t,τ* = 15 s) for both scenarios of sister-forks association as a function of the time. *t* = 0 corresponds to the time of replication of the monomer. Curves correspond to an average across all the monomers and all the simulated trajectories. **C ***SD*(*t,τ* = 15 s) for a single locus (Chromosome 2, 470 kb from left telomeres) averaged over all simulated trajectories

From simulated trajectories, we first estimate the average mean squared displacement of genomic regions (MSD, see Methods) in G1 and along S-phase, for both sister-fork scenarios (Fig. 6A, Additional file 1: Fig. S20, S21, S22A). Before the onset of replication, we observe the expected dynamics for a Rabl-like organization (Fig. 6A, Additional file 1: Fig. S20A) with an experimentally observed Rouse-like dynamics [82] characterized by a diffusion exponent 0*.*5 at short and intermediate timescales [66, 83], which transitions to a more confined dynamics at larger times due to the various geometrical constraints imposed by the Rabl organization. As S-phase progresses, we observe an overall significant reduction of the diffusion constant (Fig. 6A, Additional file 1: Fig. S20C, S22A) for both scenarios (Additional file 1: Fig. S21). Interestingly, such a decrease is maximal between ∼ 15-25 min when more than 65% of chromatin has been replicated and the number of active forks is rapidly decreasing (Additional file 1: Fig. S5). At a larger time, when most of the DNA has been replicated and with only few remaining replicating forks in the system, the reduction in diffusion constant is still present.

We thus wonder about the physical origin of this global reduction in mobility. As it is strong even at times when very few, if any (Additional file 1: Fig. S22D), forks are present, it cannot be attributed to the presence of actively replicating forks. Two possibilities remain: (i) an increase in the volumic density as, in our model, it doubles during replication; and (ii) the emergence of catenated structures from the intertwining of sister chromatids (see Additional file 1: Fig. S20D for a snapshot in the 3D simulations) [25]. The emergence of intertwined structures that slow down the relative diffusion of the two sister chromatids was characterize in our previous work, employing toy models with multiple simultaneously-fired origins [25]. In particular, the merging of converging replication bubbles with random orientation in 3D may lead to catenation points which, in absence of strand-crossing, hinder chromatin diffusion [25]. To test these hypotheses, we allow all chromosomes to replicate except one (chromosome 8, Additional file 1: Fig. S22B), thus still doubling the overall volumic density. The MSD of the unreplicated chromosome is as in G1 while the other chromosomes exhibit a perturbed dynamics. In contrast, we allow only chromosome 8 to replicate (Additional file 1: Fig. S22C). Similarly, the MSD of the replicated chromosomes is strongly perturbed while the unreplicated chromosomes behave as in G1. This suggests that changes in volumic density—at least under physiological conditions—does not impact chromatin mobility while the presence of catenated chains produced by the replication process lows the mobility of individual loci [25].

#### Fork passage transiently reduces dynamics

As mentioned above, the general changes in *MSD* observed during S-phase are not caused by the number of active forks in the system or by the mode of sister-fork interaction. However, the nontrivial topologies at forks are expected to locally impact diffusivity [25] consistent with polymer theory of branching points [84]. We thus investigate whether some dynamic signatures of fork passage may be observed in live-imaging data. For a given monomer, along a simulated trajectory, we compute the evolution of its Squared Displacement *SD*(*t,τ* = 15 s) during *τ* = 15 s as a function of time *t* (see Methods). We then compute the mean values of squared displacements around the replication time of this locus (Fig. 6B, C and Additional file 1: Fig. S23). Averaging across all loci, we observe a significant decrease in diffusion at the moment of fork passage (*t* = 0) in both scenarios, with a more pronounced reduction for the interacting case where forks are more constrained (Fig. 6B). Remarkably, our analysis suggests that, given enough statistics, this behavior can be measured even by monitoring a single locus (Fig. 6C) in multiple cells or multiple loci in one single cell (Additional file 1: Fig. S23B), thus opening the possibility of testing this prediction experimentally using live-imaging.

## Discussion

In this study, we investigate the spatio-temporal organization of eukaryotic replication in the context of budding yeast nucleus. Our approach couples biophysical and computational modeling, with new in vivo Hi-C data in S-phase. Building on our previous work [25], we were able to contextualize our coarse-grained polymer model of replicating chromatin to the specific case of budding yeast, combining the large scale Rabl organization of chromosomes with an accurate description of the underlying 1D replication dynamics. As a result, our framework shows a very high predicting power of chromosome folding during S-phase enabling us to quantitatively investigate the 3D organization of eukaryotic replication.

Throughout this study, we address the highly debated architecture of diverging sister-forks during S-phase that may—or may not—remain tethered together [23, 24]. Remarkably, in both hypotheses of non-interacting and interacting sister forks, the formation of replication bubbles leads to the local (10s of kb) reorganization of chromosomes around origins of replication. In the former case, a mild contact enrichment around early replicating origins is predicted due to entropic effects [25]. In the latter scenario, distinct “fountain” or “jet” patterns emerge via an effective loop extrusion process. While previous studies have observed such a signature around origins in experimental Hi-C data in yeast [25] and mammals [22], it was not demonstrated that these patterns are replication-dependent and do not emerge from other concomitant mechanisms.

Conducting new Hi-C experiments in a synchronized population of cells in early S-phase, we confirm the presence of fountains around early origins (Fig. 3A) and demonstrate that they are replication-dependent. Remarkably, the detection of such signal is strongly enhanced by our accurate classification of early origins if compared to the average signal computed using currently known ARS positions (Additional file 1: Fig. S3, S24). More importantly, we show that fountains were fully retained even in early-S cells depleted of cohesins, therefore excluding potential biases arising from the concomitant cohesin-mediated loop-extrusion activity and sister chromatid cohesion [57, 59–61]. However, the enrichment of contacts observed in in vivo data does not have the same magnitude as in the model prediction in the interacting scenario. We explore a number of realistic conditions—such as the radius of capture, heterogeneity in the cell population and mixed scenarios of associated and non-associated forks (Additional file 1: Fig.S9, S13) which may reduce the strength of the simulated fountain-like signal and make it closer to the experiments. All these analyses suggest that, in order to simulate fountain patterns comparable to experimental data, a significant fraction of sister-forks in vivo may not be interacting. This may be due to the limited stability of such an interaction or to the existence of unknown limiting factors that restrict the number of possible sister-fork interactions. Therefore, having the possibility to experimentally perturb sister-forks colocalization as well as monitoring the relative sister chromatid organization using Sister-C [52] during the S-phase would provide a direct mechanistic proof of the fountain formation. According to our predictions, such a perturbation should lead to a transition from a loop-extruding pattern (fountains) to a milder entropically driven compaction (typical signal in the non-interacting case). Over the years, several studies have proposed Ctf4 as a putative driver of sister-fork oligomerization [20, 22, 79]. In this study, we present the first Hi-C experiment on Ctf4-deficient cells in S-phase. We still observe fountain-like patterns (Fig. 3A), suggesting that there is no significant change in the ability of sister-forks to interact, and thus challenging the role of Ctf4 in this process. We cannot rule out the existence of compensatory mechanisms mediated by other replisome proteins that may stabilize sister-fork interactions in the absence of Ctf4. For example, components of the replisome that exhibit liquid–liquid phase-separation properties, like Ctd1 [85, 86], may be good candidates as driving factors of sister-fork interactions [31].

While we have demonstrated that standard Hi-C is able to capture replication-dependent fountain-like signals, isolating the 3D contacts made specifically by replicating DNA would likely increase their visibility and improve their quantitative interpretation. In particular, using the Repli-Hi-C technique as done in mammals by Liu et al*.* [22] or HiChIP targeting components of the replisome, could corroborate our results. While these data are not currently available in yeast, our model allows to predict Repli-Hi-C- and HiChIP-like maps in silico (Additional file 1: Fig. S25) that—consistently with what was observed in mammals [22]—exhibit stronger fountains patterns in the sisterfork interacting scenario while very mild effect in the non-interacting case. Another key finding by Liu et al*.* in the Repli-Hi-C study is the presence of fountains at termination sites (defined of type II in the original paper), suggesting a potential coordination between two convergent forks. Such patterns were not detected around termination sites in our experimental Hi-C data and in our simulations (Additional file 1: Fig. S26), even if it would be intriguing to interrogate more precisely with Repli-Hi-C if faint type II fountains may be present in yeast or if they are specific to mammals. The absence of type II fountains in yeast implies that the trivial 3D colocalization between neighboring replicons due to stochastic encounters is not sufficient for forming such patterns, and more complex mechanisms may take place at termination sites.

An interesting advantage of our framework is the ability to go beyond bulk properties and inspect large-scale structures. In particular, thanks to our minimal model of the Rabl-like organization, we were able to investigate the coupling between the Replication Timing Program and the underlying nuclear architecture, providing the first in silico evidence of a redistribution of forks across the nucleus from one pole to the other (Fig. 4, Additional file 1: Fig. S15, Additional file 5: Video S3, Additional file 6: Video S4). Our framework suggests that the previously proposed concept of “replication wave” [57] can be detected at the population level. On the other hand, we also highlight how at the single cell level, the stochasticity of chromatin dynamics and the replication timing program starts to dominate, making more challenging to directly visualize such redistribution (fainter “waves” in Additional file 5: Video S3, Additional file 6: Video S4). Interestingly, rather than a gradual shift of forks density in time away from the SPB, we observe their redistribution from the regions around SPB in early-S phase to the whole accessible part of the nucleus, consistently with the average DNA density, in mid S-phase and finally to the equatorial plane in late S-phase (Additional file 1: Fig. S15 A, B). Interestingly, we observe a transient impact of the replication on the overall local chromatin density in the interacting scenario (Fig. 4 A) via an enrichment close to SPB in early-S, which may be interrogated experimentally in the future.

Given the specificity of the Rabl organization and the abundance of forks close to the SPB in early S-phase, we question whether the passive coupling between nuclear architecture and Replication Timing program could recapitulate the experimentally observed higher-order assembly of forks into Replication Foci by Saner et al*.* in yeast [20]. In this hypothesis, the detection of larger RFi is explained by the random collisions of diffusing forks (or sister-forks), physically constrained by the underlying 3D chromosome folding, as proposed in the mammalian context by more recent studies [21, 32]. However, our in silico estimation, regardless of the interacting scenario for sister-forks, cannot recapitulate the high probability of having RFi of intermediate sizes (containing 2 to 5 sister-forks pairs) observed in super-resolution experiments. Even when introducing non-specific fork-fork interactions, we could only stabilize larger clusters without improving our prediction at intermediate sizes (Fig. 5D, Additional file 1: Fig. S18). This incompatibility might be partially explained by the challenging comparison between simulation and experimental outputs. Indeed, experimental results depend on an approximate estimate of the total number of forks at the time of measurements made by Saner et al*.* and assume that the poorer resolution on the z-axis does not introduce significant bias in cluster detection. However, we show that misevaluation of the fork numbers or misidentification of small and large clusters can quantitatively affect the outcome of the analysis (Additional file 1: Fig. S19). Recent advances in microscopy technologies that allow to reach resolutions comparable to our model [32] would enable for a more straightforward comparison in future studies. Discrepancies between simulations and experiments may also originate from missing mechanisms in the modeling framework such as cohesin-mediated loop extrusion that may facilitate the clustering of nearby replisomes along chromosomes or the selective clustering of early origins mediated by the transcription factor Forkhead that has been shown to promote the firing of about 70–100 early origins and to create preferential 3D interactions between them [47]. Moreover, potential stalling of forks at termination, which was not included in the model, could also account for the detection of larger clusters.

To fully characterize the spatio-temporal impact of replication, our model was instrumental in exploring the dynamics of replicating chromosomes at a genome-wide level. We predict a general decrease in chromatin mobility that progressively occurs during S-phase due to the emerging catenation of the two SCs [25] rather than the presence or absence of forks or changes in volumic density (Additional file 1: Fig. S22). It would be intriguing to test this hypothesis with live-cell imaging in synchronized cells during S-phase in the absence of cohesins to prevent confounding effects from cohesin-dependent loop extrusion [87] or cohesion. Moreover, such experiments would give insights into the possible role of Topoisomerase in vivo in releasing the intertwining between SCs. Locally, we quantitatively demonstrate that fork passage slows down dynamics and that this can be monitored experimentally if both the displacement and the replication status of a given genomic locus are simultaneously recorded (Fig. 6B, C) as done previously [19, 20]. By aligning measured trajectories with the time of replication (Additional file 1: Fig. S23A), our simulations suggest that given enough cells (∼ 100 s) a decrease in dynamics may be observed at the time of replication and that the magnitude of this decrease is informative on the structure of the replisome.

Naturally, our minimal coarse-grained model cannot capture the full complexity of the system. In particular, similar to other modeling studies [64–68], we aim at recovering the core features of the Rabl organization rather than a full accurate description, for example, of telomeric and centromeric regions. This limitation is illustrated by our poorer prediction for the last points of the *P*(*s*) curves (Additional file 1: Fig. S2) or the predicted faster dynamics for telomeres (Additional file 1: Fig. S20B). This is likely different from the complex behavior observed in vivo, where telomeres can be found both at the periphery and at the center of the nucleus and can aggregate through binding of the Sir complex and have slower mobility [38, 41, 83]. In order to simplify our implementation, we have also chosen an intermediate nucleus radius (1 µm) throughout replication, thus neglecting the experimentally observed gradual increase of size during S-phase [72]. Overall, we expect these limitations to have minimal impact on our results, mainly focused on early-S replication when less than 20% of the genome has been replicated.

Furthermore, to limit possible ambiguity and focus on replication-driven rearrangement of chromatin folding, we did not integrate possible interplays with SMCs. For this very reason, we performed Hi-C experiments on Scc1depleted cells to isolate replication-dependent effects on 3D structure from the drastic rearrangements mediated by the cohesin complex in S-phase [51, 52, 57, 59–61]. Given the now well-established relevance of SMCs activity for genome maintenance and function across the tree of life and along the cell cycle [3, 88, 89], it is crucial to investigate how it interferes with the core process of DNA replication. In the context of eukaryotes, despite recent advancement in the field [22, 49, 55, 59, 90, 91], it remains highly elusive to decipher how replication forks and different pools of cohesins, such as loop-extruding or cohesive, coexist in S-phase. In this perspective, our model could be instrumental in exploring the possible mechanisms for cohesion establishment upon fork passage [50, 51, 53, 54]. Similarly, equipped with loop-extrusion dynamics [92, 93] along with replication [33], our model could provide mechanistic insights on the 3D effect of cohesin stalling [59], bypassing or detaching upon fork encounters.

## Conclusions

Overall, our study suggests a significant impact of the replication process on the spatio-temporal organization of the genome at multiple scales through a loop extrusion-like process mediated by sister-fork interactions and possibly through the formation of replication foci. In yeast, where replication bubbles remain small, such an impact is transient and is not predicted to be persistent during G2/M. However, in species with larger genomes and replicon sizes [22], which may relax more slowly, we might expect a possible structural memory of the S-phase throughout G2. Future works could employ our formalism to test this hypothesis in the context of mammalian genomes where a lot of data on the genome structure and on replication are available. For example, the implemented 1D replication dynamics can be easily adapted using the results of Arbona et al*.* [63] to model human replication, thus opening exciting perspectives for applying our framework to address mechanistically the non-trivial interplay between replication and complex 3D structural features such as TADs and compartments [7–9, 11, 13–15].

What could be the biological functions of sister-fork interactions or replication foci on DNA replication itself? This question is still elusive as perturbing experimentally such processes and observing the consequences are still challenging. Integrating in our modeling framework a feedback of the 3D chromatin organization on the 1D replication dynamics via, for example, the 3D dependency of firing rates of origins or fork progressions would allow to test different mechanistic hypotheses [31, 94].

## Methods

### Experimental data

#### Haploid S. cerevisiae strains

Genotypes of *Saccharomyces cerevisiae,* (W303 RAD5 + background) strains are listed in Additional file 2: Table S3. The *ctf4::KanMX* mutation has been obtained by transformation of a PCR fragment amplified from a KanMX cassette-containing plasmid using primers 5’-GTTTCCTGAATACGCCAACATATGGGAACATATAG ATTAAATTAATAAGAAAGCTTGGGAcggatccccgggttaattaa-3’ and 5’-TTGAACGATGATTTGAACAAATGAA.

CAGGTATCAAATAATTGTCTCTTGCGTATATATATgcgcgttggccgattcatta-3’ [95]. The construct *his3::pADH1OsTIR1-9Myc::HIS3* for OsTir1 E3-ubiquitin ligase expression, as well as the Scc1-V5-AID and Cdc45-FlagX5-AID constructs have been described previously [56, 96].

#### Culture media and growth conditions

Cells arrested in G1 with alpha-factor in YPD medium (1% yeast extract, 2% peptone, 2% glucose) at 30 °C were washed 3 times with 200 mL of pre-warmed YPD and released in S-phase at 30 °C for 20 min prior to crosslinking. For Scc1-AID depletion and Cdc45-AID depletion, 2 mM IAA was added 2 h prior to G1 arrest, and in all following wash and culture media. Note that the release from G1 synchronization is not immediate. Staging cells by flow-cytometry every 5 min for 30 min after release (data not shown) showed that 20 min is an adequate early S-phase time point.

#### Hi-C

Hi-C was conducted as described in [97] with minor modifications. Briefly, ∼ 1*.*5 · 10^9^ haploid cells were fixed with 3% formaldehyde (Sigma-Aldrich, cat. F8775) for 30 min at RT with orbital agitation at 120 rpm. Formaldehyde was quenched with 330 mM glycine for 20 min at RT at 120 rpm. Cells were washed twice with cold water at 3*,*000 g for 10 min. Pellets were split inside two tubes and frozen at − 80 °C. Cell pellets (7*.*5·10^8^ cells) were thawed in ice, transferred to Precellys VK05 tube, and lysed for 3 × 30 s at 6*,*800 rpm. Between 2 and 5·10^7^ cells were processed for Hi-C using the Arima Hi-C + kit (Arima Genomics, cat. A410079) following manufacturers’ instructions. The Arima Hi-C + kit employs a dual restriction digestion (DpnII and HinfI) yielding a median fragment length of 108 bp in *S. cerevisiae*. DNA was fragmented into 300 − 400 bp fragments using Covaris M220 sonicator. Preparation of the libraries for paired-end sequencing on an Illumina platform was performed using the Thermofisher Collibri ES DNA Library Prep Kit for Illumina Systems with UD indexes (cat. A38606024) following manufacturer’s instructions. The library was amplified in triplicate PCR reactions using oligonucleotides corresponding to the Illumina sequence adaptors (5’AATGATACGGCGACCACCGAGATCTACAC-3’ and 5’-CAAGCAGAAGACGGCATACGAGAT-3’) and Phusion DNA polymerase (New England Biolabs, cat. M0531) for 11 cycles. PCR products were purified with AMPure XP beads (Beckman-Coulter, cat. A63881) and resuspended in pure H2O. The Hi-C library is quantified using the Qubit DNA high sensitivity kit (Thermo Scientific, cat. Q32851) on a Qubit 2 fluorometer (Thermo Scientific, cat. Q32866). Library quality control, paired-end sequencing (2 × 150 bp) on Illumina NovaSeq6000 or NovaSeq X Plus, and data QC were performed by Novogene UK. Libraries used are listed in Additional file 2: Table S4. The numbers of biological replicates for Hi-C in each investigated condition are provided in Additional file 2: Table S4.

#### Hi-C read alignment and data normalization

For each condition, paired-end reads from different biological replicates were catenated, aligned and contact data filtered using the Hicstuff [98] pipeline function in “cutsite” mode with a quality threshold of 20. Briefly, pairs of reads are aligned iteratively and independently using Bowtie2 in its most sensitive mode to the *S. cerevisiae* reference genome R64-2–1, obtained from the https://www.yeastgenome.org website. Each uniquely mapped read was assigned to a restriction fragment. Contacts were filtered as described in [99] and PCR duplicates were discarded. Data were binned at 1 kb using Hicstuff rebin function and converted to a cooler format with Hicstuff convert. Contact maps are finally normalized using the function *balance.iterative correction* (ICE algorithm) from the cooltools library (v 0.5.1) [100].

#### Flow-cytometry

Approximately 10^7^ cells were collected by centrifugation, re-suspended in 70% ethanol and fixed at 4 °C for at least 24 h. Cells were pelleted, re-suspended in 1 mL of 50 mM sodium citrate pH 7*.*0 and sonicated 10 s on a Bioruptor. After washing, cells were treated with 200 µg of RNase A (Euromedex, cat.9707-C) at 37 °C overnight. Cells were then washed and incubated for 30 min with 1 mL of 50 mM sodium citrate pH 7*.*0 with 16 µg of propidium iodide (Fisher Scientific, 11425392). Flow cytometry profiles were obtained on a MACSQuant machine and analyzed using Flowing Software 2.5.1.

#### Protein extraction and western blotting

Protein extraction and western blotting were performed as described in [91]. Protein extracts for western blot were prepared from 5*.*107 to 108 cells. Cells were lysed in cold NaOH buffer (1*.*85 N NaOH, 7*.*5% v/v beta-Mercaptoethanol) for 10 min in ice. Proteins were precipitated upon addition of trichloroacetic acid (15% final) for 10 min in ice. After centrifugation at 15*,*000 g for 5 min, the pellets were re-suspended in 100*µ*L of SB + + buffer (180 mM Tris–HCl pH 6*.*8, 6*.*7 M Urea, 4*.*2% SDS, 80 *µ*M EDTA, 1*.*5% v/v Beta-mercaptoethanol, 12*.*5 µM Bromophenol blue). Proteins were denatured upon heating 5 min at 65 °C. Pre-cleared extracts were resolved on 12% precast polyacrylamide gel (Bio-Rad, cat. 4,561,043) and blotted on a PVDF membrane (GE Healthcare, cat. 10,600,023). Membranes were probed with mouse anti-AID antibody (clone 1E4, CliniSciences, M214-3) diluted at 1: 1000 for Cdc45-Flag-AID, an anti-GAPDH antibody (Invitrogen, MA515738) diluted at 1: 10,000, or an anti-V5/Pk1 monoclonal antibody (Fisher Scientific, cat. R960-25) diluted at 1: 10,000 for Scc1-V5-AID. Primary antibodies were revealed with an HRP-conjugated anti-mouse IgG antibody diluted at 1: 10,000 (Abcam, ab6789) using Immobilon Forte western HRP substrate (Merck, WBLUF0100) and a Chemidoc MP Imaging system (BioRad).

### Polymer model and simulations

#### Null model

Each chromosome is modeled as a self-avoiding walk on a fcc lattice composed of *N* beads and dynamically evolving via a Kinetic Monte Carlo (KMC) algorithm similar to other studies [25, 101–105]. In addition to excluded volume, monomers are subjected to a standard potential to account for the chain bending rigidity:

1 $${H}_{bend}=\kappa {\sum }_{i=2}^{L-1}\left(1-\text{cos}{\theta }_{i}\right)$$

with *θ*_*i*_ being the angle between monomers *i* − 1, *i* and *i* + 1 and *κ* the bending modulus in kT units. To simulate chromatin, we consider standard values for the fiber diameter (*σ* = 20 nm) and compaction (50 bp/nm) [66], recovering an approximate bead size of 1000 bp and Kuhn length of 100 nm (*κ* = 3*.*217 kT). Time mapping between the simulation Monte Carlo Steps (MCS) and real time is done by computing the Mean Squared Displacement (MSD) of a monomer ⟨((*r*(*t* + *τ*) − *r*(*t*))^2^⟩ as a function of the time step *τ*. For this analysis, we used a single chromosome simulation of chromosome 4 of *Saccharomyces cerevisae* (polymer chain of 1531 monomers) in periodic boundary conditions and a 5% volumic fraction. Similarly to previous studies [25, 103, 106, 107], we then compared it to the experimentally-observed ${MSD}_{exp}\left(\tau \right)\left[\mu {m}^{2}\right]\approx 0.01{\tau }^{1/2}$ with *τ* in seconds, measured in *Saccharomyces cerevisae* [83], leading to 1 MCS = 0*.*075 ms.

#### Full genome implementation

##### Initialization

Similarly to previous analogous implementations [64, 66–68], we model the nuclear envelope by introducing a spherical barrier to monomers in the system. In the lattice framework used here, this is accomplished by occupying all sites above a certain radius from the box center, now inaccessible to the polymer chains due to excluded volume interactions (Additional file 1: Fig. S1A). Based on microscopy experiments [41, 108], we set the diameter of the sphere to 2* µm* which corresponds to ≈ 70 lattice units given a bead size of 20 nm (Additional file 1: Fig. S1B). We then introduce several distinct polymer chains into the system to model each individual chromosome. Since we aim to model the haploid budding yeast genome, the number of chains is set to 17 where the 12th and 13th chains both model chromosome 12 (see below). Each chain is built using the HedgeHog algorithm [103, 109]: the chain is recursively grown to the desired size (different for each chromosome), starting from a simple backbone of length *L/*4 where *L* = 70 is the box size. Arbitrarily, we choose the backbone to be divided into two equally long linear branches (each *L/*8), in random directions (See Additional file 1: Fig. S1A for an example). The starting position of each backbone (corresponding to the first monomer of the chain) is placed at one of the 17 points inside the equatorial plane. These points are produced using the sunflower algorithm [110] to efficiently pack *n* points inside a circle (Additional file 1: Fig. S1A, small panel). To randomize the process and avoid any bias, chromosomes are built in a random order, ensuring that the two random directions used to build the backbone of a new chain do not cross the ones already initialized. Note that such initial configuration does not aim to mimic any biological scenario (such as chromatids organization upon Mitotic exit). The choice of a such randomized V-shaped backbone allows for simple and efficient construction of non-crossing chromosomes in the lattice, whose chain ends (telomeres) are randomly oriented. Given the strong forces that will be introduced into the system to recreate the Rabl configuration, this initial choice is likely to have a minimal impact. In fact, different initialization strategies in other studies [70] eventually converge to similar results once those forces are established.

##### Rabl organization

To mimic centromere attachment to the SPB, we introduce a spring-like potential between the centromere of each chromosome and position ${\overrightarrow{x}}_{SPB}=(\frac{L}{2},\frac{L}{2},L)$ in XYZ coordinates (Additional file 1: Fig. S1B). This forces the centromeres in the first times of the simulation to move toward the SPB (spring constant *k* = 100 kT). We complement our potential to allow the centromeres to freely diffuse in a spherical shell located between 250 and 325 nm from the SPB. The Hamiltonian describing the kinetics of centromeric monomers is as follows:

2 $$H_{centromere}=\begin{cases} 0 & \text{if } \ 250\ nm <|\vec{x}-\vec{x}_{SPB}|<325\ nm \\ +k(|\vec{x}-\vec{x}_{SPB}|)^2 & \text{otherwise}. \end{cases}$$

To simulate the tethering of telomeres to the nuclear envelope (NE), we implement an outward force acting on the terminal monomers of the chains, with a negative spring potential connecting them to the centers of the spheres ${\overrightarrow{x}}_{center}=(\frac{L}{2},\frac{L}{2},\frac{L}{2})$ to push them towards the NE. Again, we allow the telomeres to freely diffuse within a distance of 50 nm from the spherical wall (Additional file 1: Fig. S1B). Therefore, the Hamiltonian describing the kinetics of telomeric monomers is as follows:

3 $$H_{telomere}=\begin{cases} -k(|\vec{x}-\vec{x}_{center}|)^2 & \text{if } |\vec{x}-\vec{x}_{center}|<950\ nm \\ 0 & \text{otherwise}. \end{cases}$$

Since the nucleolus consists of a large physical barrier for non-rDNA, we introduce an additional impermeable wall positioned at 800 nm in the z-direction (Additional file 1: Fig. S1B,C). Spherical confinement and the nucleolus wall lead to a final volumic fraction Φ = 3%, compatible with the chromatin concentration standardly used for modeling yeast chromosomes [103].

##### Modeling chromosome 12

To simulate chromosome 12 and account for its role in the nucleolus assembly, we split chromosome 12 into two polymer chains anchored by one side to the nucleolus wall (Additional file 1: Fig. S1C). The first chain models the first 460 kb of chromosome 12 where its rightmost monomer corresponds to a boundary of rDNA (Additional file 1: Fig. S1C). The remaining portion of the chromosome, without centromere, is modeled with a second chain whose leftmost monomer constitutes the second boundary of rDNA, while the rightmost one is the second telomere. The two rDNA beads are pulled to the nucleolus wall with a spring potential only in the z-direction (downward) enabling their localization at the nucleolus wall periphery:

4 $$H_{rDNA}=kz^2$$

Similar to other implementations [68, 69], no constraint was applied to the 3D distances between the two rDNA boundaries, which are allowed to diffuse independently on the nucleolus surface.

#### Self-replicating polymer

##### Generic model

We model explicitly replication in 3D using a specialized class of self-replicating polymers to simulate each chromosome. On-lattice chain duplication in KMC simulations was physically characterized in detail in our previous study [25]. Although, in this study, we include multiple chains and the Rabl-like organization, the local realization of replication is analogous since each polymer chain, while governed by a global underlying 1D replication dynamics (see below), duplicates “independently” in 3D. Briefly, a given unreplicated monomer can be used as an origin of replication, leading to the formation of a replication bubble. In particular, origin firing consists of introducing a new monomer into the system, connecting it to the two neighbors of an origin along the maternal chains. This process effectively creates two polymer branches that locally simulate the two nascent SCs. Monomers with triple connectivity at the extremities of the replication bubbles are defined as replication forks with a given directionality based on their position with respect to the origin. With a constant speed *v* = 2*.*2 kb/min, every fork in the system can trigger further replication by introducing additional monomers at the fork positions and moving the triple connectivity. In particular, we convert this physiological speed into lattice units which gives a probability of ∼ 3 · 10^−6^ for a given fork to be replicated per MCS. Both in the case of origin firing and fork progression, the newly added monomers are positioned at the same site of their homologous beads, thus temporarily breaking the excluded volume rule. As monomers diffuse rapidly in 3D, such a constraint is quickly reinstated. A more detailed description of the local realization of monomer duplication in the lattice can be found in Fig. S1 of our previous study [25]. Replication bubbles merge once two forks of opposite directionalities converge along the chain. In such a configuration, the two converging forks, now neighbors along the chain, are always replicated in pairs, leading to the formation of a single larger bubble. Similarly, when a fork is adjacent to a telomere, the bubble is opened by replicating both the fork and the chain-end monomers.

##### 1D replication dynamics

We introduce implicit 1D replication dynamics by adapting the formalism of Arbona et al*.* [62, 63] to our KMC framework. Replication is modeled as a bimolecular reaction between a limited number of *N*_*f*_ firing factors and the assigned origins of replication and a firing trial move is introduced in our scheme. Under the assumption of a well-mixed system, at a given time, a set of *N*_*origin*_ origins is randomly selected (see below) and each origin of this set is fired with a probability:

5 $${\text{Prob}}_{\text{firing}}=\left({N}_{f}-\frac{{N}_{forks}\left(t\right)}{2}\right)\cdot OriginRate$$

where (N_*f*_ −N_*forks*_(*t*)/2) represents the current number of available freely diffusing firing factors and *OriginRate* is a constant rate, equivalent of k_*on*_ in [62, 63]. To model the heterogeneous time patterns of eukaryotic replication, the set of N_*origins*_ is sampled from the “Initiation Probability Landscape Signal” (IPLS) computed in [63]. The IPLS is a signal that defines origin priming efficiency and was inferred in [63] through a machine learning approach to accurately reproduce experimental Mean Replication Timing and Replication Forks Directionality data [44]. Practically, the sampling of origins is performed by randomly picking a fixed number of monomers (N_*origins*_ = L_*chromosome*_/5 as inferred by Arbona et al. in [63]), the weighted probability of each monomer being given by the binned IPLS at the same resolution of our model (1 kb). Note that the drawing is performed with replacement, which means that each monomer can be selected multiple times to account for that a certain coarse-grained genomic position may contain multiple (fine-scale) origins, all of which have the same probability of firing (*OriginRate*).We then set* N*_*f*_= 120 and *OriginRate* = 1 · 10^−9^*MC*^−1^ which are the KMC parameters equivalent to the ones inferred by Arbona et al*.* in [63]. Firing factors are rapidly introduced in the system once the S-phase starts according to the formula *N*_*f*_(1 − 0*.*13exp^−*t/τ*^) with *τ* = 1 min and 30 s.

##### Interactions during S-phase

Co-localization of sister-forks is maintained by adding an elastic-like potential between the forks to the Hamiltonian of the system on which the Metropolis criterion is applied:

6 $${H}_{tot}={H}_{bend}+{H}_{sister-forks}$$

With

7 $${H}_{sister-forks}=-{J}_{sister}\sum_{\left(i,j\right)\in \{sister-forks\}}\left[1+\Theta \left({d}_{i,j}-th\right)\left(\frac{{d}_{i,j}^{2}}{{th}^{2}}-1\right)\right]$$

where the sum is performed over all the pairs of still active sister forks localized at monomers *i* and *j*, *d*_*i,j*_ is the 3D Euclidean distance between *i* and *j*, Θ(*x*) is the Heaviside step function and *th* = 50 nm is the distance below which the energy saturates to *J*_*sister*_ = 100 kT. Springs between forks are kept until one of the two interacting partners is lost due to the merging of replication bubbles or the replication of the chain-ends. In other words, the two remaining forks after the encounter freely diffuse in 3D any binding energy connecting them.

##### Non-specific forks interactions

We include contact interactions with strength *J*_*f*_ between all replication forks in the system. Similarly to previous copolymer lattice models [102–104, 106], we include an additional term on the Hamiltonian of the system:

8 $${H}_{forks}=-{J}_{f}\sum_{i,j}{f}_{i,j}{\sigma }_{i}{\sigma }_{j}$$

where *σ*_*i*_ = 1 if monomer *i* is a replication fork, otherwise *σ*_*i*_ = 0. *f*_*i,j*_ = 1 if monomers *i* and *j* are first or second nearest-neighbors otherwise *f*_*i,j*_ = 0. In practice, during the KMC algorithm, we keep track for each lattice site on the number of forks within a 40 nm distance. This number is locally updated after each trial move (see scheme in Additional file 1: Fig. S15). This strategy allows for a rapid scanning for nearby forks and facilitates Hamiltonian evaluation. Similarly to other implementations [102–104, 106], we do not impose any constraints on the number of interactions for a given fork.

The value of *J*_*f*_ = 1.7 kT was established by visually inspecting Hi-C maps (Additional file 1: Fig. S17A) and offdiagonal aggregate plots (Additional file 1: Fig. S17B) between early origins for increasing values of *J*_*f*_. In particular, we selected an energy value that lies in the transition between non-aggregated and aggregated states [102, 104].

### Data analysis

#### Computation of Hi-C maps

The contact probability between two monomers *i* and *j* is defined as the probability that the 3D Euclidean distance *d*_*i,j*_ between *i* and *j* is less than a fixed radius of contact *r*_*c*_. *r*_*c*_ is set to 80 nm in all figures except Additional file 1: Fig. S2A. The full genome contact matrix is then computed by ordering all of the monomers according to their genomic sequence. The resulting matrix is loaded into a cooler file [100] at 1 kb resolution (monomer size) and then converted to a multi-resolution*.mcool* file to obtain the maps at lower resolution. In the presence of replicating DNA, we do not distinguish between SCs when populating the in silico Hi-C maps. As a result, similarly to the in vivo experiments, the contacts between two genomic positions *i* and *j* can be populated by different pairs of monomers according to the current replication status of the two. Unless stated otherwise, contact maps (either in silico or in vivo) are always normalized using the function *balance.iterative* *correction* (ICE algorithm) from the cooltools library (v 0.5.1) [100]. Such normalization is commonly used to obtain equal visibility for all genomic loci and correct for sequence specific amplifications and copy-number variations [111]. In the case of Hi-C maps of partially replicated genomes, the ICE algorithm also corrects for the relative increase of contacts at replication bubbles with respect to unreplicated DNA, simply arising due to sequence duplication (See Additional file 1: Fig. S27). Expected contacts in *cis* (*P*(*s*) curves) and in *trans* are computed using the *expected* functions of cooltools [100]. Pileup plots are computed using the “pileup” function in coolpup library [112]. Specifications on the number of independent simulations and frames used for each plot are summarized in Additional file 2: Table S2.

To define early and late replicating origins, we used the IPLS from [63] (Additional file 1: Fig S3) that codes for the frequency of firing in our framework. We use the Scipy function *find peak* [113] to extract the IPLS peaks. We define the 15% highest peaks (signal > 5) as early origins of replication and all remaining peaks whose signal is above 1 as late origins. Similarly, CARs positions used in Additional file 1: Fig S9B, were defining using the Scipy function *find* *peak* (height threshold = 20) [113] on the MCd1p (cohesin subunit) ChIP-Seq profile from [61].

#### Comparison between in silico and in vivo signals

##### Comparison with in vivo Hi-C maps in G1

In Fig. 1B, we illustrate the main features of the Rabl organization at the full genome (16-kb resolution, top panel) and single chromosome (1-kb resolution, Bottom panel) levels. To plot together in vivo and in silico maps, we rescale the simulated contact matrices by multiplying the signal by a factor $\gamma =\frac{{P}_{exp}(s=16kb)}{{P}_{sim}(s=16kb)}$. The Pearson correlation between predicted and experimental maps at the 16-kb resolution is 0*.*93.

We also compute Pearson correlations for the 16 individual chromosome maps at 1 kb resolution (Additional file 2: Table S1), giving an average of 0*.*88.

We compute aggregate plots around centromeres (on diagonal plots, see below for technical details) normalizing by the intra-chromosome *P*(*s*) (Fig. 1D). Our model correctly predicts the average “flame” shape around centromeres in experimental maps even if we observe a too strong depletion of contacts between centromeres and chromosome arms, compared to in vivo maps (blue stripes in Fig. 1D).

We then quantify the enrichment of centromere-centromere (*trans*) contacts arising from the imposed colocalization of centromeres in the confining shell around SPB by computing off-diagonal aggregate plots. To compare experiments and simulations, we normalize both by dividing by the respective *P*_*intra*_(*s* = 10 kb) (Fig. 1C). This strategy allows to recover the relative enrichment with respect to the intra-chromosomal contacts. In particular, we choose a scale above the polymer persistence length where our simulations match well the in vivo signal (see Additional file 1: Fig. S2). In the simulations, we estimate that the average centromere-centromere contact frequency is ∼ 11% of the *P*_*intra*_(*s* = 10 kb) while the experiment exhibits a more moderate enrichment ∼ 8%.

In both cases, these quantitative differences are likely due to the strong modeling assumptions made. For instance, chromosomes are strictly bound by the strong potential to localize between 250 and 325 nm from the SPB. Relaxing these conditions should decrease the centromere-centromere contact frequency. Similarly, the strong potential brings telomeres and centromeres strictly to different parts of the nucleus (NE and SPB respectively). This polarity is enforced in all trajectories, leading to a very strong depletion of contacts between centromeres and chromosome arms (blue stripes in Fig. 1A). The segregation between centromeres and telomeres might be more heterogeneous in vivo and other biological processes (such as condensin activity) may hinder this effect.

##### Comparison with in vivo P(s) curves

To compare simulated and experimental *P*(*s*) in Additional file 1: Fig. S2B,C and S13B, we multiply simulated curves by a factor *α*, which minimizes the Mean Squared Error (*MSE*) within a given genomic interval [*s*_*min*_*,s*_*max*_]:

9 $$MSE=\frac{1}{{s}_{max}-{s}_{min}}{\sum }_{s={s}_{min}}^{{s}_{max}}{\left({P}_{exp}\left(s\right)-\alpha {P}_{sim}\left(s\right)\right)}^{2}$$

Practically we use *s*_*min*_ = 10 kb and *s*_*max*_ = 1 Mb when comparing *P*(*s*) averaged among different chromosomes (Additional file 1: Fig. S2B and S13B) while from *s*_*min*_ = 10 kb and *s*_*max*_ = 50 kb in the case of individual chromosomes (Additional file 1: Fig. S2C).

##### Comparison between on-diagonal aggregate plots

When comparing on-diagonal aggregate plots in different simulated conditions or between in vivo and in silico data, no additional normalization was used. In fact, for these particular analyses (such as in Fig. 1D or Figs. 2 and 3), we present Observed over Expected maps where each pixel (*i,j*) is normalized by the average contact frequency at the corresponding genomic distance *s* =|*i* − *j*| (*P*(*s*) signal).

#### Spatial distribution of forks in the nucleus

In Fig. 4 and Additional file 1: Fig. S15 we plot the probability distribution function of forks as a function of the distance from the SPB. Summing over all the trajectories, we count the number of forks at a distance between *r* and *r* + *dr* where *dr* = 5 nm and at a given time window. We then normalize by the total number of forks and the corresponding volume of the slice: *V* (*r* + *dr*) − *V* (*r*) where *V* (*r*) = (3*R* − *r*)*r*^2^*π/*3 is the spherical cap for a distance *r* from the SPB and *R* = 1000 nm is the radius of the sphere. We also compute the same observable for all the monomers to assess whether the localization of forks follows the average polymer organization.

#### Analysis of RFi

##### Definition of RFi in silico

We define RFi in silico by constructing a graph based on the 3D positions of forks, $\{{\overrightarrow{r}}_{i}\}$, at each simulation frame. For a given threshold 3D distance *θ*, we define an association matrix *A*_*i,j*_, where *A*_*i,j*_ = 1 if |${\overrightarrow{r}}_{i}$− ${\overrightarrow{r}}_{j}$|≤ *θ* and *A*_*i,j*_ = 0 otherwise. An RFi is defined as an independent component of *A*_*i,j*_ (i.e. a set of forks where each fork in the set is separated by a distance > *θ* from any forks outside the set). The total number of RFis corresponds to the number of graph components. To efficiently compute this quantity, we use the Python library *networkx* [114].

##### Comparison with experimental data

To compare our predictions with the experimental estimates of forks within RFi by Saner et al*.* [20] (Fig. 5D), we adapt our cluster detection to mirror the experimental protocol. In the original paper [20], the authors analyze 3D microscopy images of replicating yeast nuclei where PCNA (forks subunit) has been labeled with GFP proteins. Each distinct bright spot detected is then classified as an individual RFi and the number of forks inside is computed on the basis of an in silico estimation of 302 forks in the nucleus at that specific replication stage. In practice, the relative intensity of each focus (signal of the focus divided by total signal) is multiplied by the total number and cast strictly to an even number. This assumption is motivated by early microscopy evidence that sister-forks are associated [19]. Experimental estimate of forks pairs within each RFi (gray squares) was extracted from Fig. 3E of the original paper [20]. In our in silico predictions, we roughly follow a similar approach by dividing the number of forks of each RFi by the total number of forks in the system in that specific frame to obtain a relative signal. For each RFi, we then multiply this value by the average number of forks between *t* = 2 min and 30 s and *t* = 3 min 45 s (229 forks). Finally, the resulting number was made even. In Additional file 1: Fig. S19, we explore confounding factors that may impact the classification.

#### Analysis of chromosome dynamics

In order to simplify the analysis, all the observables described below are computed for only one chromatid for each chromosome. In particular, once a given monomer is replicated, each measurement is computed only for its copy belonging to the Sister Chromatid 1.

##### Mean squared displacement

In Fig. 6, Additional file 1: Fig. S20C,S21 and S22, we compute the Mean Squared Displacement with the formula:

10 $$MSD\left(\tau \right)={\langle {\left(\overrightarrow{r}\left(t+\tau \right)-\overrightarrow{r}(t)\right)}^{2}\rangle }_{t,traj,mon}$$

In Additional file 1: Fig. S20B, we average over the monomer (*mon*) in the centromeric or telomeric regions, defined as being at a genomic distance less than 20 kb of a centromere or telomere respectively. Similarly, in Additional file 1: Fig. S22 the MSD is computed restricting the average to monomers belonging to individual chromosomes. In Additional file 1: Fig. S22B, we impair the replication of chromosome 8 by manually deleting all the potential origins before the onset of replication. In contrast, “instantaneous” replication was achieved by setting a homogeneous high firing rate for all origins and high replication speed (Additional file 1: Fig. S22C) for all chromosomes or exclusively for chromosome 8 (and hindering replication for all the others, Additional file 1: Fig. S22D).

##### Time-rescaled squared displacement

The average Squared Displacement at 15 s in Fig. 6B, is computed with the formula:

11 $$SD\left(t,\tau =15 sec\right)={\langle {\left(\overrightarrow{r}\left(t+\tau \right)-\overrightarrow{r}(t)\right)}^{2}\rangle }_{traj,mon}$$

To rescale the time, we recorded each individual *SD* trajectory along with its replication status and aligned all trajectories at the moment of replication (*t* = 0 min) (see Additional file 1: Fig. S23A for an example of a single monomer in a single simulation).

## Supplementary Information

Additional file 1. Supplementary figures S1-S27 and captions of supplementary videos S1-S4.

Additional file 2. Supplementary tables S1-S4.

Additional file 3. Supplementary video S1.

Additional file 4. Supplementary video S2.

Additional file 5. Supplementary video S3.

Additional file 6. Supplementary video S4.

## Data Availability

Simulation code is available at https://github.com/physical-biology-of-chromatin/LatticePoly [121] on the branch named *Yeast full genome*. The source code for the specific branch used in this study was deposited on Zenodo (doi:10.5281/zenodo.17417149) [122] under a CC BY-NC 4.0 open-source license. Hi-C data are available at the GEO repository numbers indicated in Additional file 2: Table S4. New HiC data produced for this study have been deposited at GEO repository number (GSE309730) [123]. Published HiC data for G1-arrested [58] and metaphase-arrest (*cdc20*) [97] cells can be found at GEO repository numbers GSE179641 [124] and GSE272969 [125] respectively. The ChIP-Seq profile of Mcd1p from [61] was downloaded as bigwig file from GEO (GSE151416) [126].
