# Supplementary material for: Genome-wide modeling of DNA replication in space and time confirms the emergence of replication specific patterns in vivo in eukaryotes
Source: Genome Biol. 2025 Dec 22;26:431. doi: 10.1186/s13059-025-03872-4 (PMC12723920; doi:10.1186/s13059-025-03872-4)
Supplement: Supplementary file 1 — Additional file 1. Supplementary figures S1-S27 and captions of supplementary videos S1-S4. [file 13059_2025_3872_MOESM1_ESM.pdf]

Additional File 1
Genome-wide modeling of DNA replication in space and time confirms the emergence of replication specific patterns in vivo

Dario D'Asaro, Jean-Michel Arbona, Vinciane Piveteau,
Aurèle Piazza, Cédric Vaillant, Daniel Jost

This document contains:

- Supplementary Figures S1 to S27
- Captions of Supplementary Videos S1 to S4

Supplementary figures

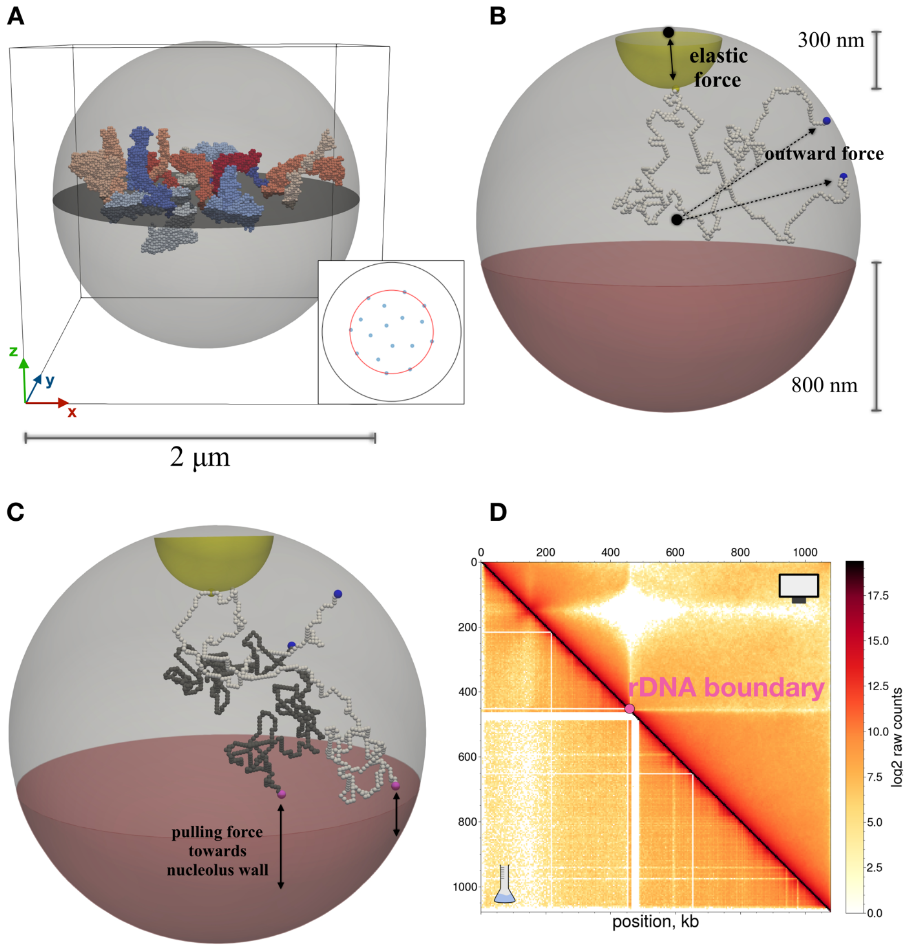

Fig. S1: **On lattice implementation of the yeast Rabl organization.** (A) Example of an initialized configuration on the lattice. The gray area indicates the volume accessible to monomers. (Inset) The starting monomer of each chromosome for the hedgehog algorithm is positioned in one of the blue points within the red circle located at the equatorial plane. Yellow lines illustrate for two chromosomes the "v-shaped" backbone used to recursively grow the polymer chain. (B) Example of a chromosome in a Rabl configuration. The yellow monomer indicates the centromere confined in the yellow shell to mimic SPB attachment. Blue monomers correspond to the telomeres pushed towards the NE. The red area indicates the nucleolus, whose volume is assumed to be inaccessible to monomers. When considering all the chromosomes, we recover the brush-like architecture schematized in the inset panel, where several polymer branches (the 32 arms) radiated from the SPB surface. Compared to a non-constrained linear polymer, inter-arms contacts are enriched (see Fig 1 B,C).*(Continue in next page)*.

Fig. S1: *(Continued)*(C) Example of a configuration of chromosome 12 on the lattice. The light gray polymer chain models the first 460 kb of DNA. The rDNA boundaries (indicated by pink monomers) are pulled toward the $-\vec{z}$ direction. The darker polymer chain models the rest of Chromosome 12, i.e. the part that does not contain the centromere. (D) Comparison between experimental and simulated contact maps for chromosome 12.

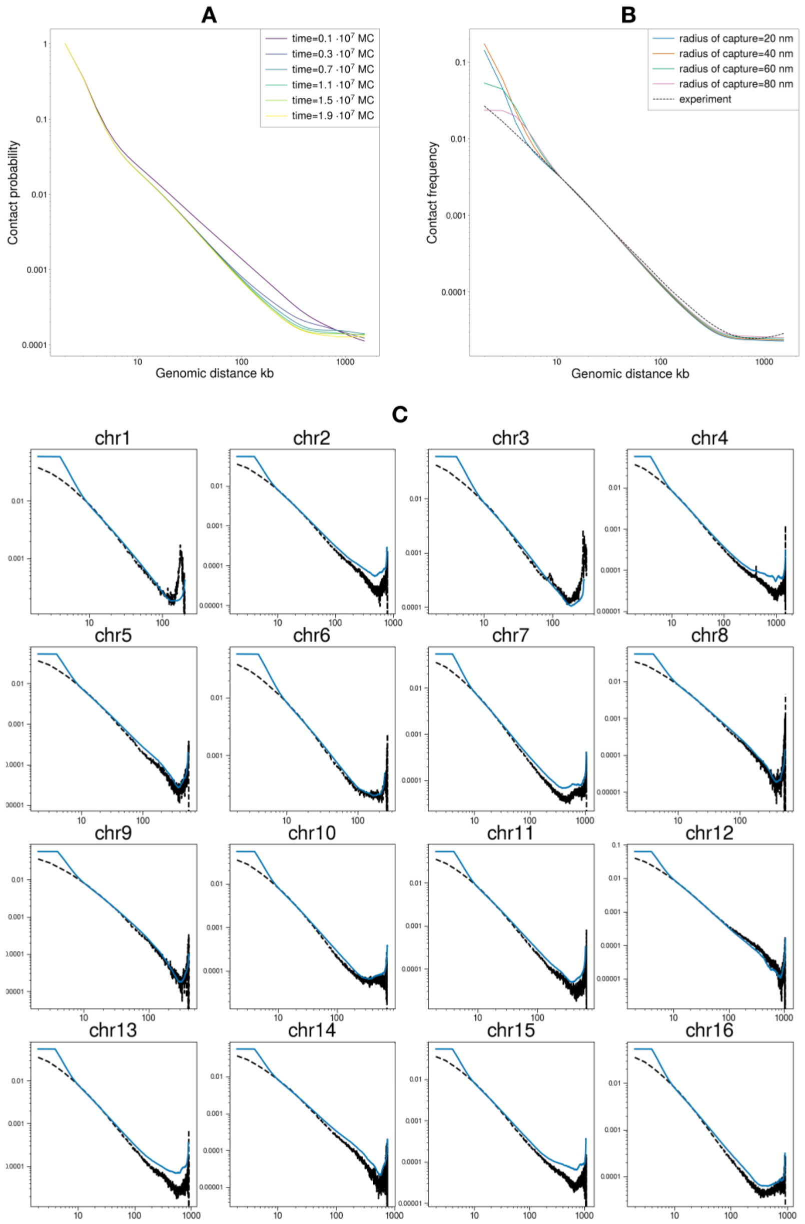

Fig. S2: **Comparison with *in vivo P(s)* curves.**(A) Since the initial configuration in Fig. S1A does not aim to capture any biological phenomenon, we need to establish a relaxation time required to reach a G1-like 3D chromosome organization. While centromeric, rDNA and telomeric monomers reach their constrained locations in a few MC steps, each chromosome has to equilibrate its internal configuration before making any measurement. *(Continue in next page)*.

Fig. S2: *(Continued)* For this reason, we investigate intra-chromosome $P(s)$ by computing contact maps at various time steps of the relaxation process (radius of contact of 40 nm) establishing a relaxation time of 10^7 MCS. The $P(s)$ signal was smoothed and averaged between all chromosomes using *cooltools* functions. In this case, no normalization is applied (raw contacts). (B) $P(s)$ curves for 4 different radii of capture (20, 40, 60,80 nm) after averaging over all the chromosomes. Simulated curves are rescaled by multiplying by a constant factor α computed as described in the Methods (s_{min} = 10 kb and s_{max} = 1 Mb). Interestingly, independently of the chosen radius of capture, our *in silico* curves well recapitulate the experimental scaling of intra-chromosome $P(s)$ observed in G1 HiC experiment. However, our model does not capture very well the contact frequency at very large scales. In particular, in the smoothed experimental data (Fig. S2B black dashed curve), we can observe a small increase in contact at the end of the curve due to telomere-telomere contacts.(C) Simulated (blue, $r_c = 80$ nm) and experimental (black) $P(s)$ curves for all 16 chromosomes. Simulated curves were rescaled multiplying by a constant factor α where s_{min} = 10 kb and s_{max} = 50 kb. For every chromosome, the simulations capture the increase of contact frequency at the end of the curve due to telomere clustering. However, in our simulations such an increase is much steeper (see chr1 or chr3 for more visually clear examples), likely due to the more rigid constraints imposed by our modeling. In particular, telomeric monomers are always strictly tethered to the NE, while they may also be observed in the interior [1]. We speculate that the lack of such heterogeneity in our model might result in the flat $P(s)$ at larger scales once averaging all the chromosomes and smoothing (Fig. S2B).

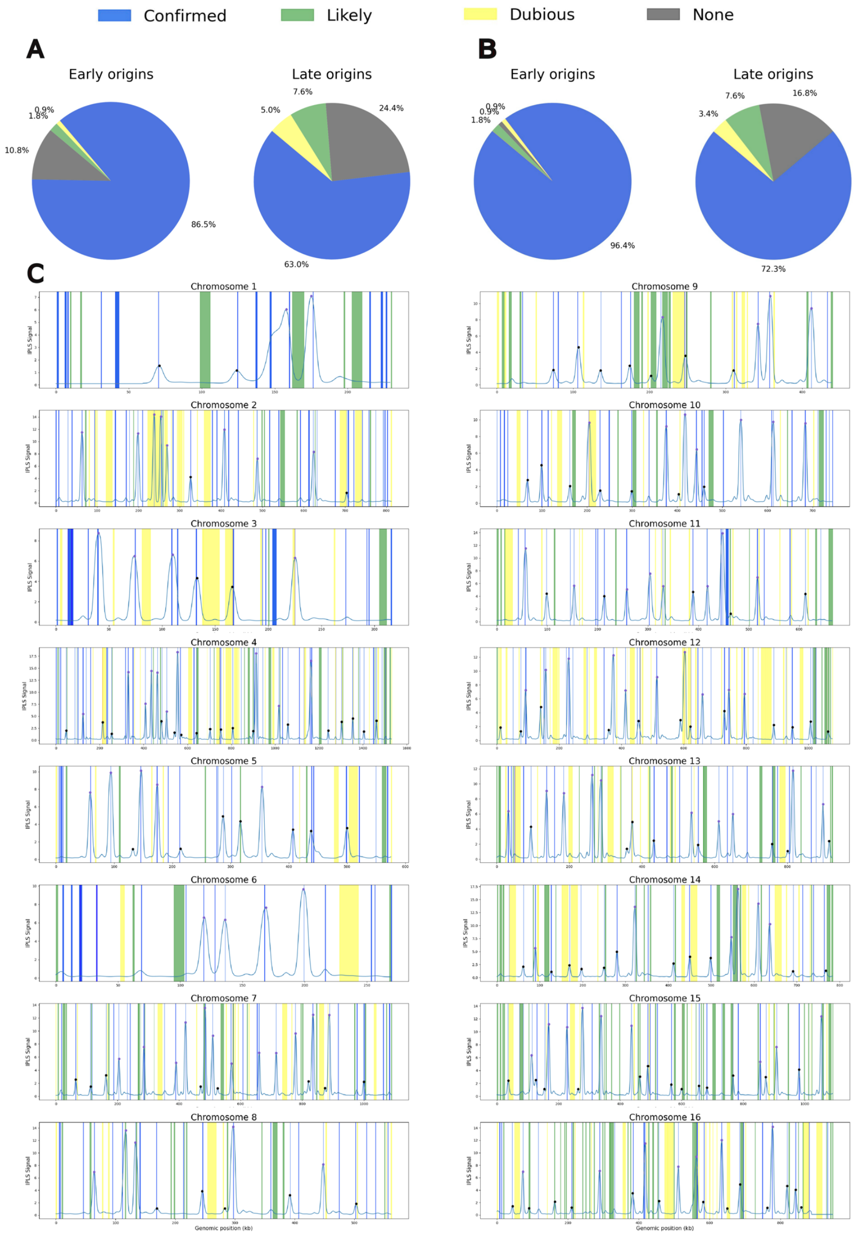

Fig. S3: *(Caption in the next page.)*

Fig. S3: *(Continued)* **Comparison with Oridb ARS.** (A,B) Percentage of origins detected through the *IPLS* (See Methods for more details) which coincide to known ARS in budding yeast from the Oridb database [2]. According to the nomenclature in Oridb, each origin corresponds to either a “Confirmed” (blue), “Likely” (green) or “Dubious” ARS or “None” if no match was found. When multiple ARS match, we use the best classification available. In (A), we assign origins to a class when the position inferred from the *IPLS* is at most 1 kb from an ARS, considering the start and end in the sequence from Oridb. On the right we used the origin in this study referred as ”Early” (see orange circles in panel B) while on the left, only “Late” origins are considered (see black circles in panel B). (B) Same as (A) but relaxing the classification with a genomic distance between our origins and ARS of 2 kb. (C) *IPLS* profiles and inferred origin positions (peaks) for all chromosomes. The positioning of ”Confirmed” (blue), “Likely” (green) or “Dubious” ARS from Oridb is also shown. In conclusion, our method is very accurate in predicting origins positioning, since a high percentage was found to correspond to ”Confirmed” ARS. Interestingly, several confirmed ARS do not match with a peak of the *IPLS* signal, which is computed solely using *in vivo* MRT and RFD data [3].

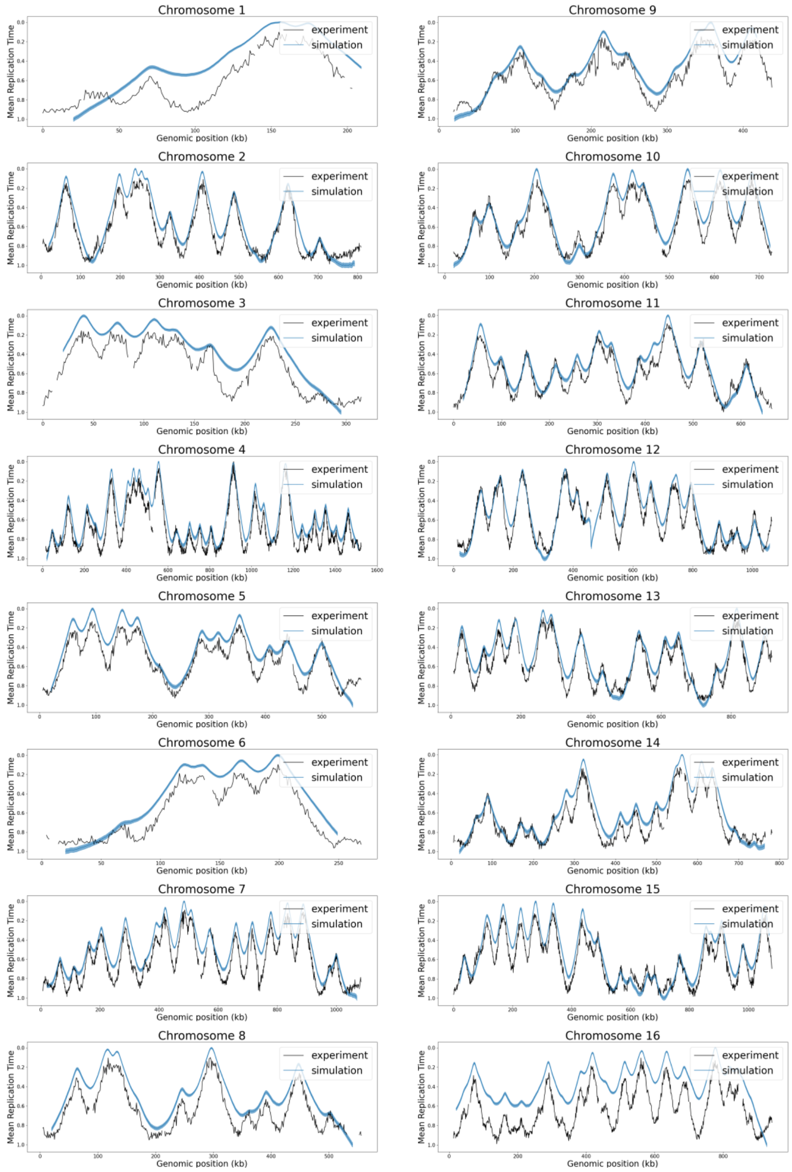

Fig. S4: **1D Replication dynamics**. Comparison between the experimental [4] (black lines) and simulated (blue lines) data for all the chromosomes. We compute the simulated MRT by rescaling the average replication time of each monomer between 0 and 1. We exclude in the computation the last 20 kb of DNA which are not well captured by the model. Slow replication of telomeric regions can strongly affect the signal once simulated MRT is rescaled as it can still be observed for chromosome 16 where we speculate that the IPLS fails to capture origin firing close to the telomeres.

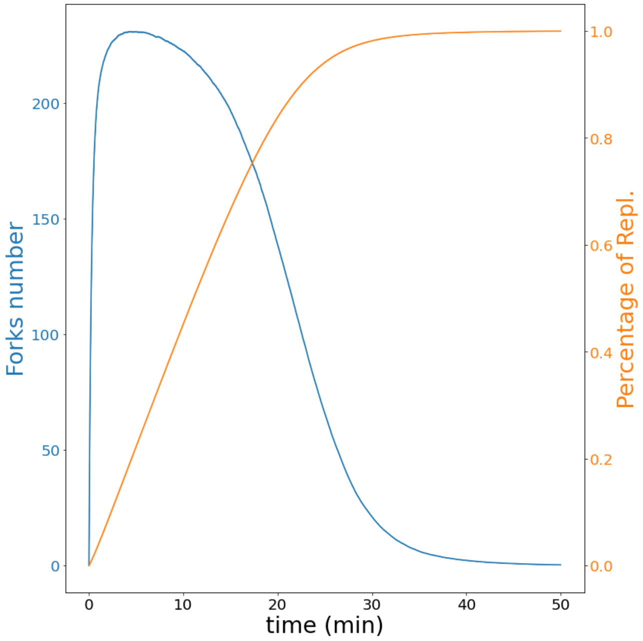

Fig. S5: **Average number of forks and percentage of replicated chromosomes over time predicted by the model over the whole genome**.

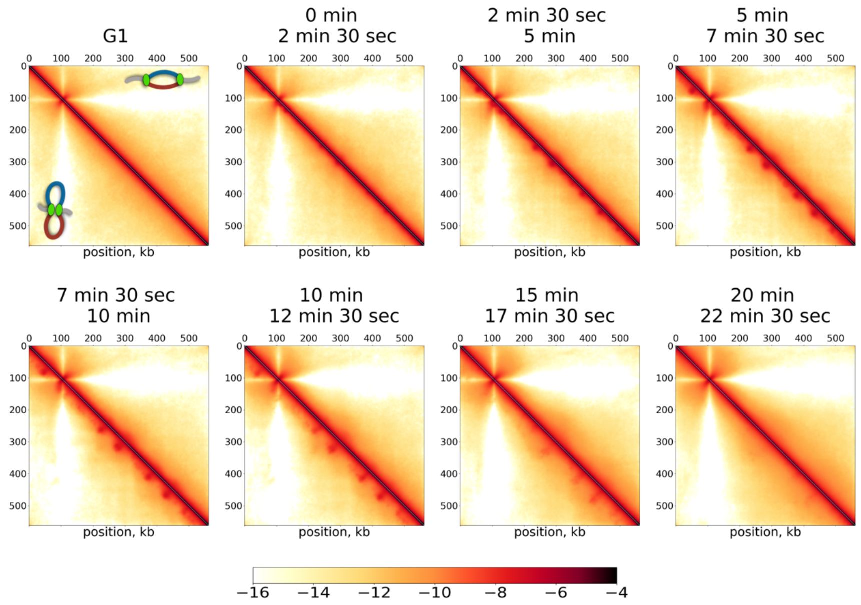

Fig. S6: Example of normalized HiC maps (chromosome 8) at different times along S-phase for interacting (bottom triangles) and non-interacting (top triangles) sister-forks.

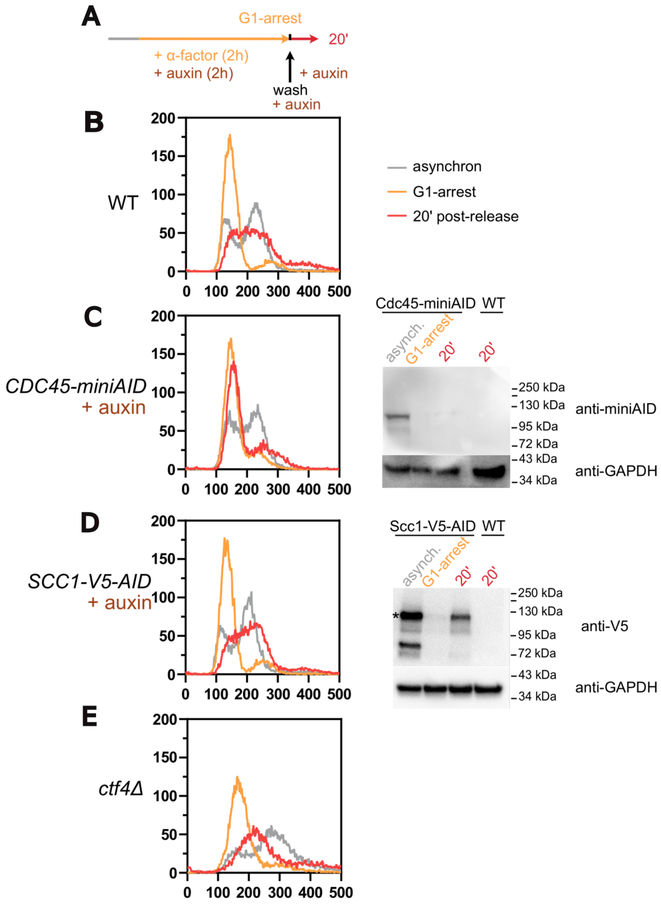

Fig. S7: **Cell-cycle progression and protein depletion of experimental Hi-C data.** A) Experimental set-up for G1-synchronisation and release, and for protein depletion. (B-E) Cell-cycle progression in samples WT (APY607) (B), Cdc45-miniAID (APY539) +auxin (C), Scc1-V5-AID (APY1696) +auxin (D) and Ctf4Δ (APY1814)(E). Western blots are shown in cases of protein depletion (C and D, right). Western blot of Cdc45-miniAID probed with an anti-miniAID antibody and the loading control GAPDH (C, right) and of Scc1-V5-AID probed with an anti-miniAID antibody and the loading control GAPDH (D, right). Expected size of Cdc45-miniAID is 82kDa and expected size of Scc1-V5-AID is 70kDa.

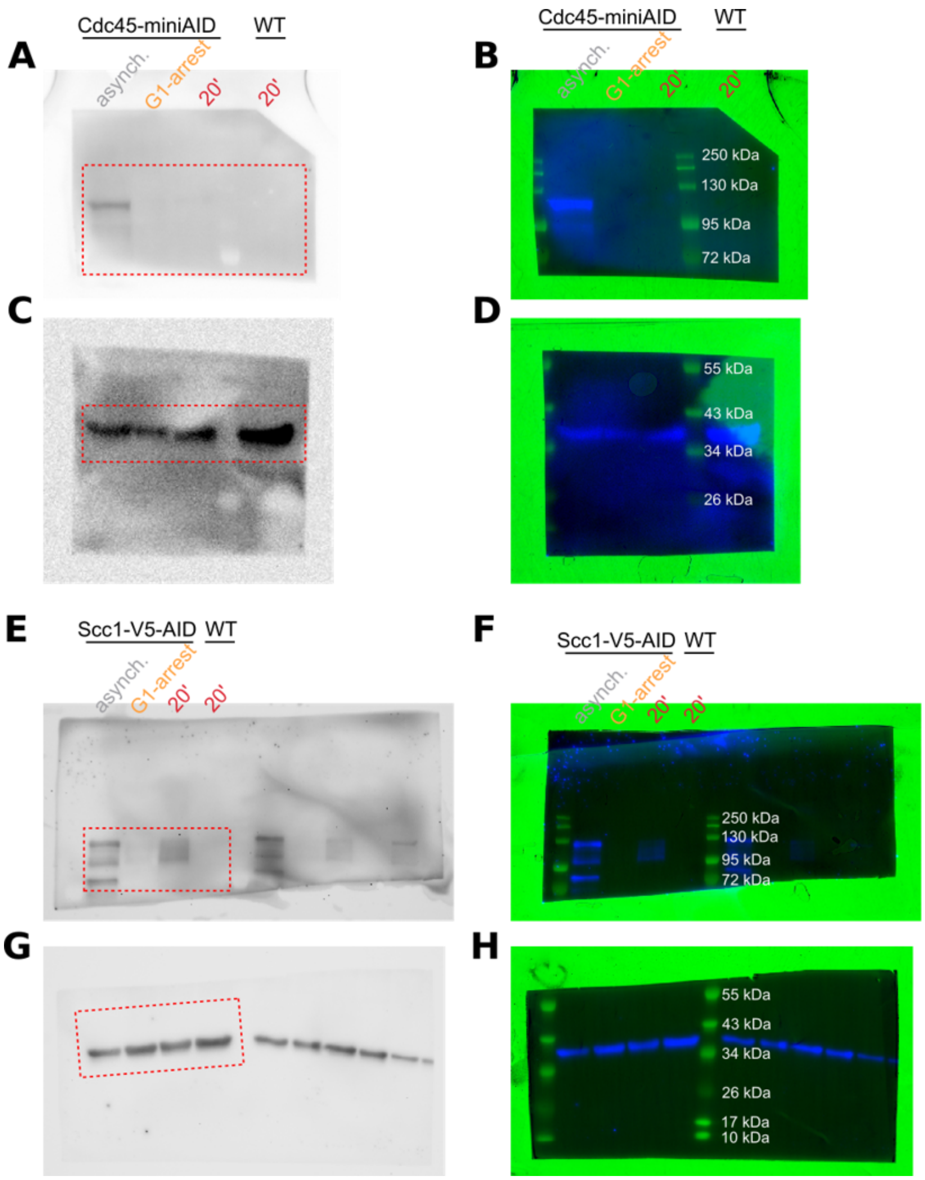

Fig. S8: **Full-length Western blots shown in Fig S7**. (A, B, C, D) Full-length Western blot of Cdc45-miniAID probed with an anti-miniAID antibody (A,B) and the loading control GAPDH probed with an anti-GAPDH antibody (C,D). (E, F, G, H) Full-length Western blot of Scc1-V5-AID probed with a V5 antibody (E, F) and the loading control GAPDH probed with an anti-GAPDH antibody (G,H). Red dotted lines indicate crops shown in Fig. S7. Chemiluminescence plots (A, C, E, G) are shown as well as composite plots showing chemiluminescence in blue and colorimetric signal in green with corresponding ladder molecular weight indicated in white (B, D, F, H).

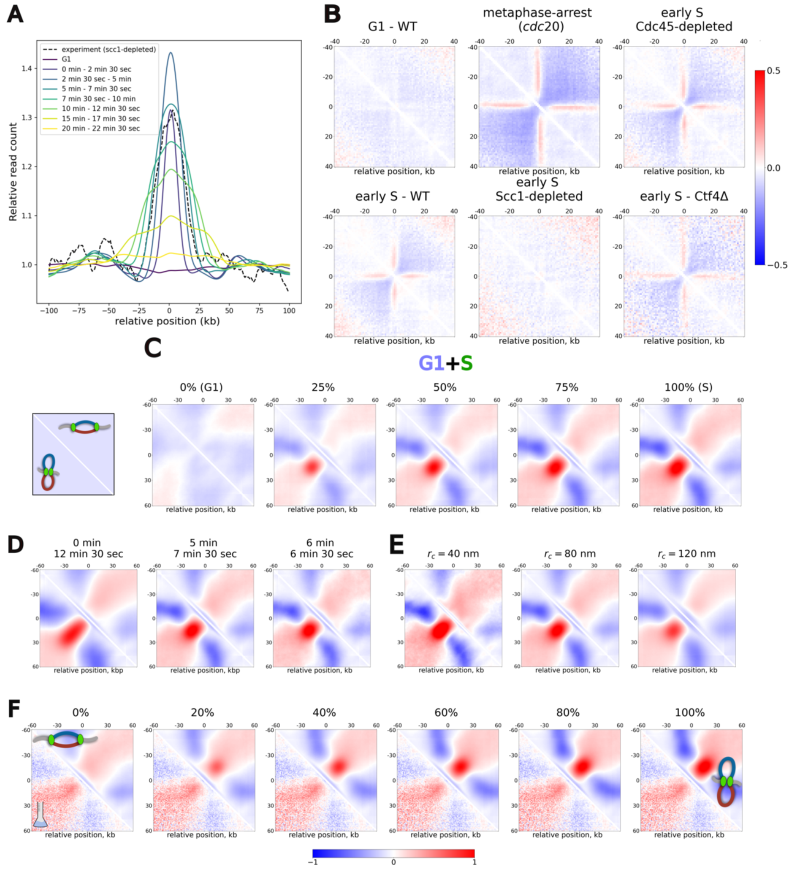

Fig. S9: (A) To identify the time in the simulations corresponding to the *in vivo* early-S Hi-C data, we compute the total read count for each genomic position in a 200 kb region around early-replicating origins. Practically, this consists in summing all the contacts of the corresponding rows of the unbalanced contact matrix. To compare experiments and simulations, we divide the signal by the average read count of regions at a distance between 50 to 100 kb from the origin (regions more likely to not being replicated). Analyzing the width of the peak (correlated with the typical replicon sizes) we find that times between $t = 5$ and $t = 7$ min and 30 sec give the best match with the experimental signal. (B) Average normalized ($\log_2$ Observed over Expected) contact maps around CARs (see Methods). The cross-like pattern, signature of CAR-CAR interactions typical of mitotic chromosomes (see *cdc20*-arrested) starts to be established in early-S for all the strains containing cohesin. (*Continue in the next page*)

Fig. S9: *(Continued)* This is observed regardless the presence (WT and Ctf4) or absence (Cdc45) of ongoing replication. Despite the partial depletion of Scc1 (see Methods), the Scc1-depleted strain does not show significant enrichment of CAR-CAR interactions anymore, similar to G1-arrested cells. (C-F) Average normalized ($\log_2$ Observed over Expected) contact maps around early replicating origins to test possible confounding factors in the enrichment intensity. Unless stated otherwise, to test different conditions, we used as the simulated map between 5 min and 7 min and 30 sec as representative of early S. Note that to generate the mixed configurations (either between replicating/G1 or between interacting/non-interacting), we always aggregate the raw contact maps first and then re-balance the final matrix. (C) Dilution of the signal due to asynchronization. Each map was computed by mixing G1-like, unreplicated trajectories with replicating ones at different percentages, for non-interacting (upper triangles) and interacting (lower triangles) sister-forks. Even with a strong dilution, the replication-dependent signals typical of each scenario are still visible. (D) Effect of time interval used to produce HiC maps during replication in the non-interacting (upper triangles) and interacting (lower triangles) sister-fork scenarios. A wider interval, due to the increased heterogeneity in replicon sizes, results into more extended fountains in the presence of sister-forks interactions and to the loss of enrichment on the diagonal in their absence. (E) Effects of the radius of capture r_c used to compute contact maps in the simulations. When r_c increases, we observe a strong loss in the intensity and shape of the enrichment.(F) Mixed configurations of interacting and non-interacting sister-fork trajectories with different percentages (upper triangles). The resulting maps are compared with the experimental aggregates for Scc1-depleted cells (lower triangles).

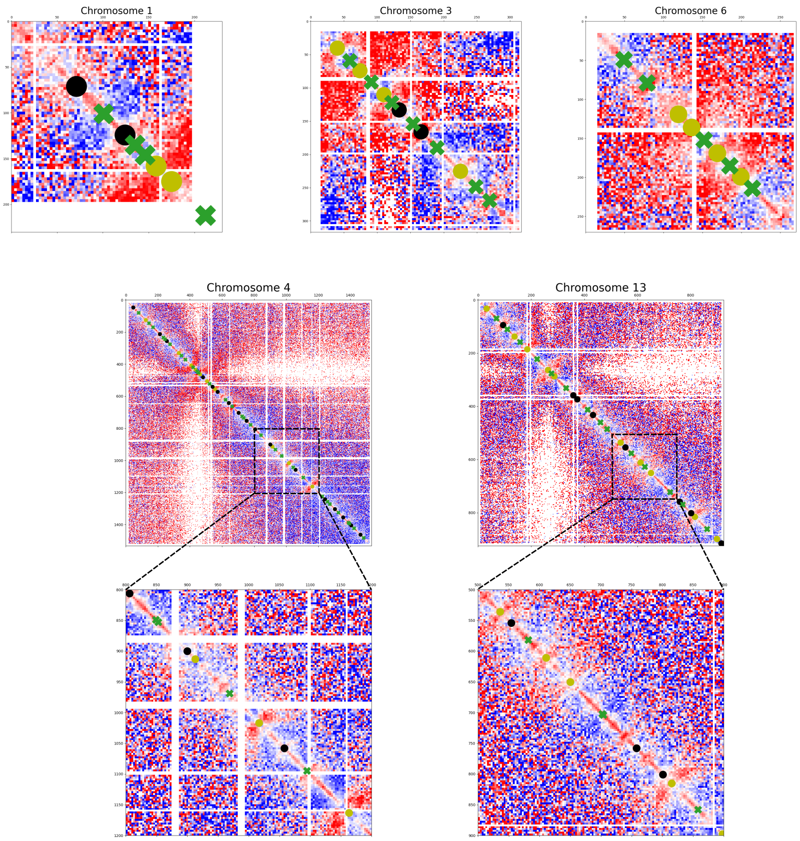

Fig. S10: **Differential maps: Scc1-depleted over G1.** $\log_2$ ratio between Scc1-depleted (early S, cohesin depleted) and G1 HiC maps for three short chromosomes (chromosome 1, 3 and 6) and two long ones (chromosome 4 and 13) with a zoom on a smaller region. Maps were plotted at 3 kb resolution. Yellow and black dots indicate early and late replicating origins respectively. Green crosses indicate termination sites which were inferred from our simulations (see Fig. S26 for more details). A clear correlation between fountain-like patterns and origins is observed.

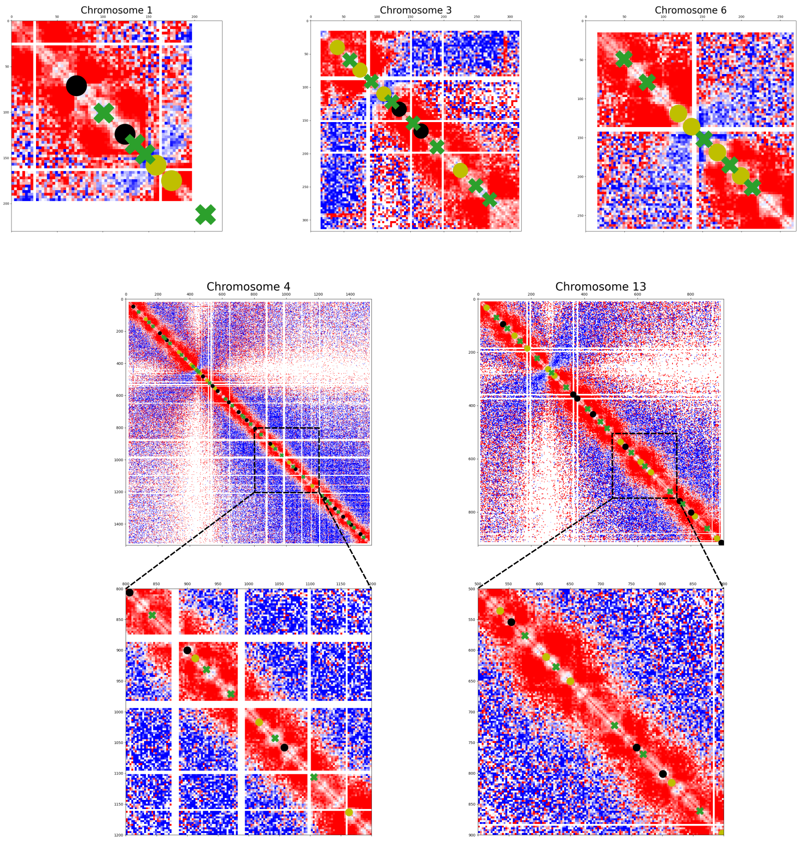

Fig. S11: **Differential maps:** ***cdc20*** **over G1.** $\log_2$ ratio between *cdc20* (metaphase arrested) and G1 HiC maps for three short chromosomes (chromosome 1,3 and 6) and two long ones (chromosome 4 and 13) with a zoom on a smaller region. Maps were plotted at 3 kb resolution. Yellow and black dots indicate early and late replicating origins respectively. Green crosses indicate termination sites which were inferred from our simulation (see Fig. S26 for more details). We observe strong, ubiquitous enrichment of contacts along the diagonal, not specifically at origins. Such enrichment can be explained by the loading of cohesin loops on SCs at this stage of the cell cycle.For this analysis only one biological replicate was used (AD266, see Additional file 2: Table S4).

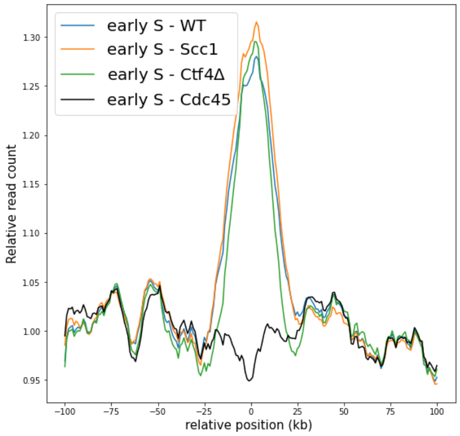

Fig. S12: **Average replicon size around early origins in different experimental conditions.** We compute the total read count for each genomic position in a 200 kb region around early-replicating origins. Practically, this consists in summing all the contacts of the corresponding rows of the unbalanced contact matrix. We then divide the signal by the average read count of regions at a distance between 50 to 100 kb from the origin (regions more likely to not being replicated). We repeat the analyses for all the experiments with cells in early S (WT, Cdc45, Scc1-deplted and Ctf4-Δ). As expected, no enrichment is found in Cdc45 mutant where SCs synthesis is impaired. WT and Scc1 exhibit slightly different peaks heights and same amplitude (replicon size). On the other hand, Ctf4-Δ cells show smaller replicons, suggesting a potential slowing down in the fork progression.

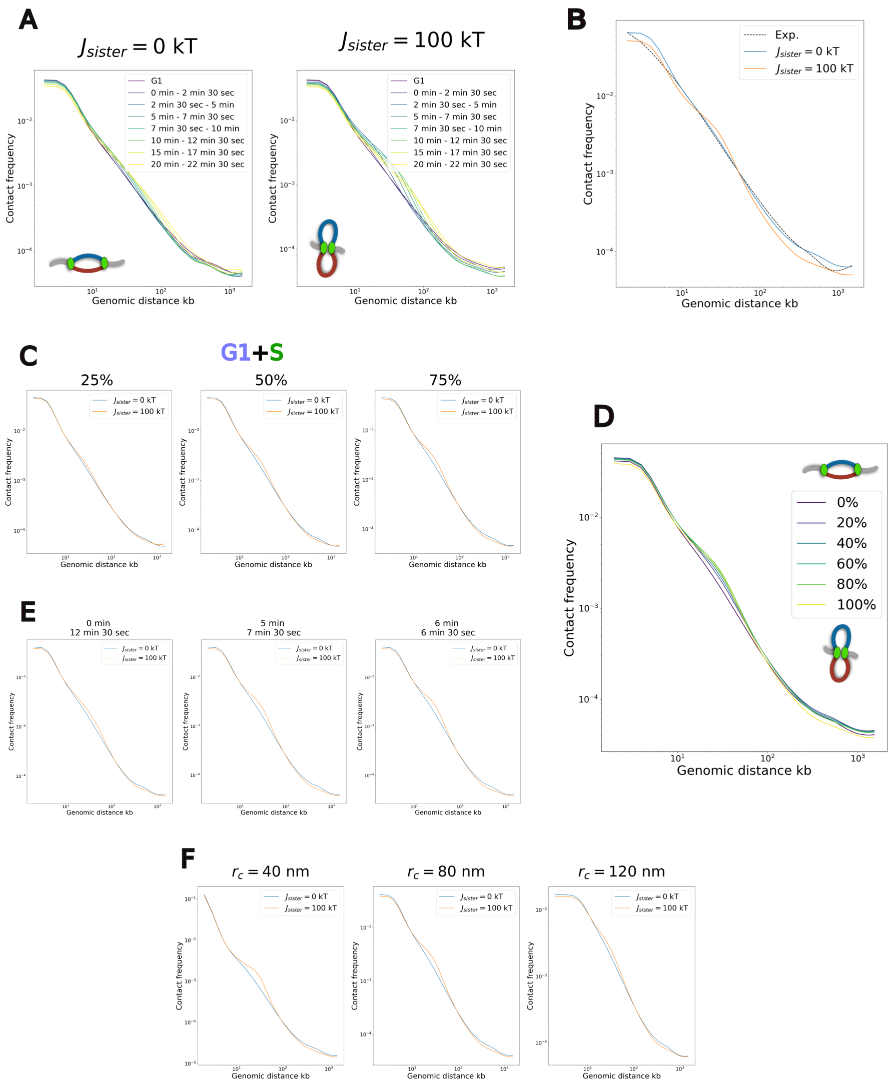

Fig. S13: ***P(s)* curves in S-phase.** (A) Time-evolution of *in silico* $P(s)$ in the two scenarios. Maps for interacting sister-forks (Rights) are characterized by a "shoulder"-like feature which evolves according to the replicon size as expected for an extrusion process. (B) Comparison between *in vivo* $P(s)$ (Scc1-depleted cells) and simulations (between 5 min and 7 min and 30 sec) for non-interacting (blue) and interacting (orange) sister-forks. Simulated curves are rescaled by multiplying by a constant factor α computed as described in the Methods ($s_{min} = 10$ kb and $s_{max} = 1$ Mb). Notably, the "shoulder" feature is not clearly observed *in vivo*. *(Continue in the next page)*

Fig. S13: *(Continued)* (C-F) $P(s)$ curves in presence of possible confounding factors as done in Fig. S9 for aggregates around early origins. Unless stated otherwise, to test different conditions, we use the map simulated between 5 min and 7 min and 30 sec as early-S map. (C) Dilution of the signal due to asynchronization. Each map was computed mixing G1-like, unreplicated trajectories with replicating ones at different percentages and for non-interacting (blue) and interacting (orange) sister-forks. (D) Mixed configurations of interacting and non-interacting sister-forks. Trajectories from interacting sister-forks simulations are mixed with non-interacting ones with different percentages (different curves). (E) Effect of time interval used to produce HiC maps during replication for non-interacting (blue) and interacting (orange) sister-forks. (G) Effects of the radius of contact r_c used to compute contact maps. The computations in (C,D,E,F) show how some of these factors could strongly impact $P(s)$ shape in the interacting case, smoothing the curve towards the non-interacting forks signal. Similarly to Fig. S9, these confounding factor could additively contribute in diluting the shoulder in the *in silico* $P(s)$ and obtain predictions more consistent with the *in vivo* curve.

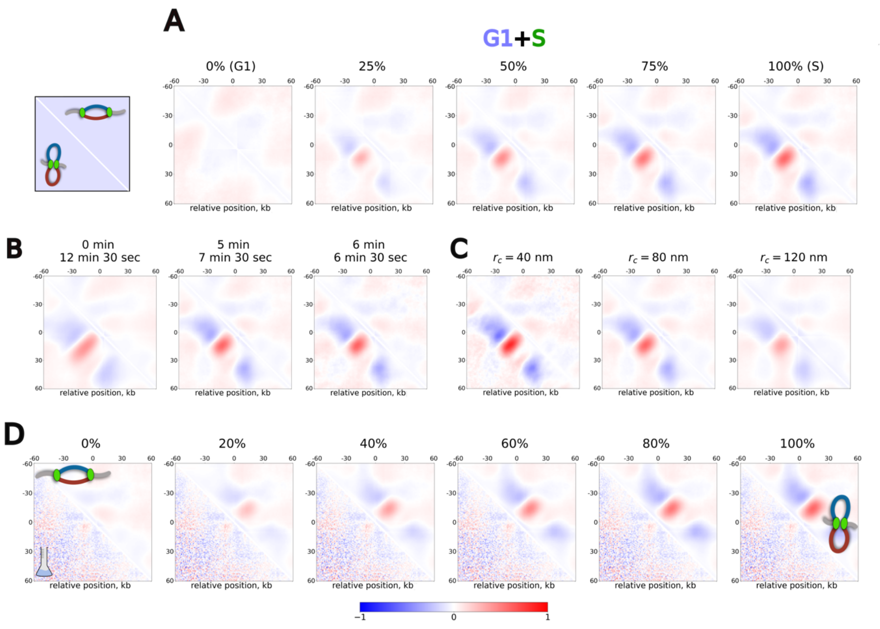

Fig. S14: **Aggregate plot around late origins.** (A-D) Average normalized ($\log_2$ Observed over Expected) contact maps around late replicating origins to test possible confounding factors in the enrichment intensity. Unless stated otherwise, to test different conditions, we used as replicating map the one between 5 min and 7 min and 30 sec. All the following results lead to analogous conclusions to the ones described in the context of early replicating origins. (A) Dilution of the signal due to asynchronization. Each map was computed mixing G1-like, unreplicated trajectories with replicating ones at different percentages and for non-interacting (upper triangles) and interacting (lower triangles) sister-forks. (B) Effect of time interval used to produce HiC maps during replication non-interacting (upper triangles) and interacting (lower triangles) sister-forks. (C) Effects of the radius of contact r_c used to compute contact maps. (D) Mixed configurations of interacting and non-interacting sister-forks. The resulting maps are compared with the experimental aggregates for Scc1-depleted cells.

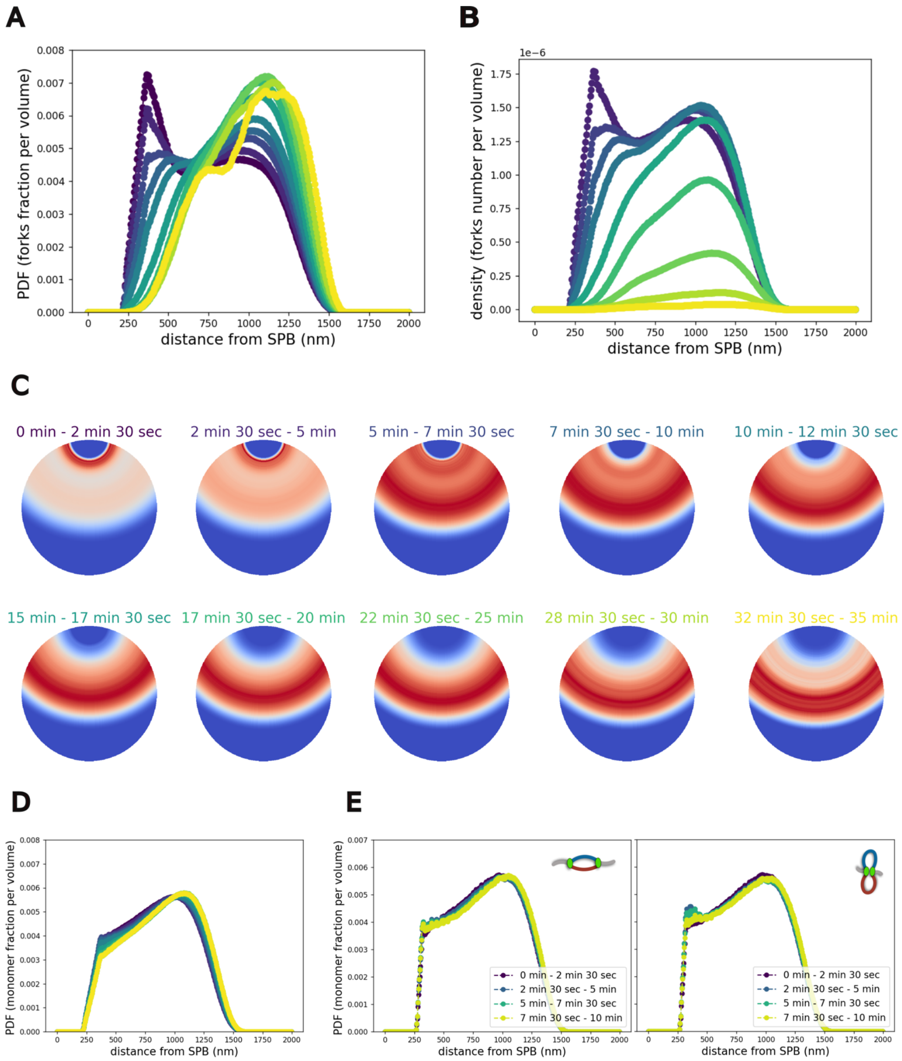

Fig. S15: **Spatial distribution of forks in longer simulations**. (A,B) Probability distribution function (PDF) of fork positionnning as a function of the distance r from the SPB (see Methods) at different time intervals covering the full S-phase (labels for each of the colors can be found in (C). Only results for the non-interacting sister-forks are presented ($J_{sister} = 0$ kT). On (A), each distribution is normalized to 1 (as in the main text in Fig.4B). On (B) we plot the density of forks without such normalization to highlight the decrease in the total number of forks as replication proceeds. (C) 2D graphical representation of the time evolution of normalized forks densities plotted in (A). This longer set of simulations shows how the remaining forks in late S-phase are enriched at the equatorial plane while the peak in density around the SPB is gradually lost. *(Continue in the next page)*

Fig. S15: *(Continued)* (D) PDF to find a monomer at a distance r from the SPB at different time intervals covering the full S-phase (labels of each of the colors can be found in (B). Minor changes in the PDF can be observed such as a slight decrease at 250 nm from the SPB (the approximate location of centromere attachment). Note that this effect is mild when compared to the forks' redistribution shown in (A). It may be due to local changes in density. (E) PDF of monomer positioning as a function of the distance r from the SPB in presence and absence of sister-forks interactions. We can observe how replication by extrusion leads to a mild enrichment in early S-phase at 250 nm from the SPB relatively to the non-interacting scenario.

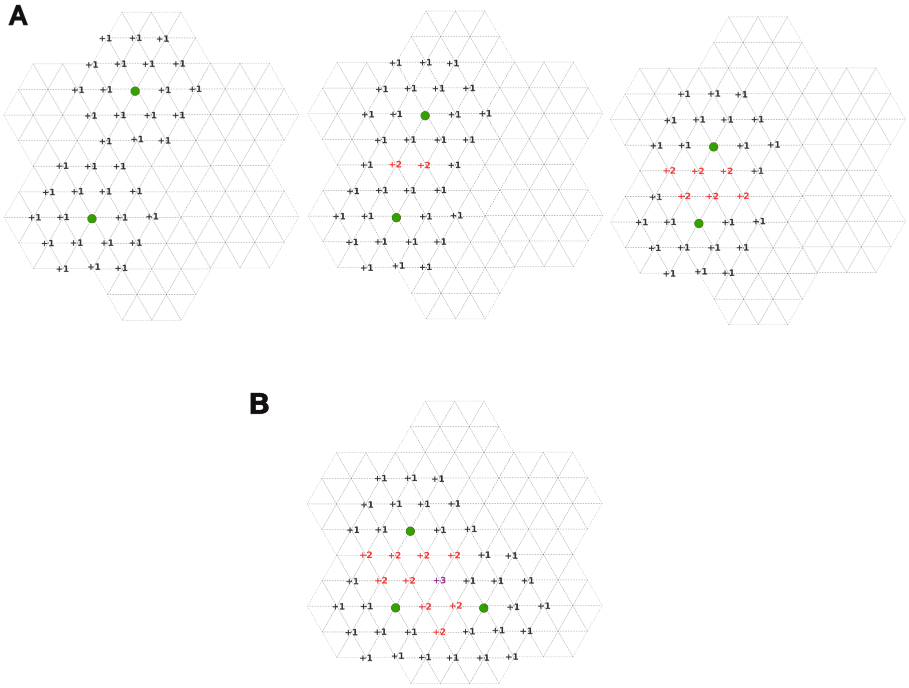

Fig. S16: **Non-specific attractive interactions between forks in the lattice.** (A) Example of how non-specific contact interactions between any pairs of fork are introduced in the lattice model (see Eq. 8 in Methods). For each fork (green circles), all the sites within 2 nearest neighbor distance have a decrease in energy +1. As two forks moves (from left to right) some sites will now will be more energetically favorable because within the interaction radius of the two. (B) Example of three forks colocalizing in space.

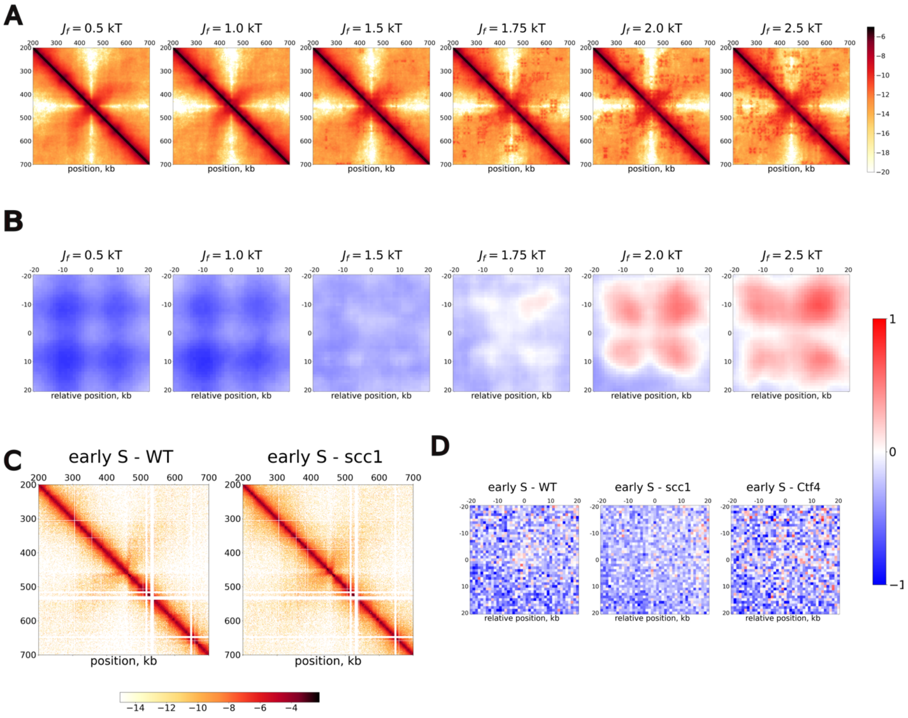

Fig. S17: **Model with non-specific attractive interactions between forks.** (A) Example of normalized HiC maps (500 kb region of chromosome 4) for increasing values of J_f in the interacting sister-fork case ($J_{sister} = 100$ kT). Interestingly, we can clearly observe a transition at $J_f > 1.5$ kT where different forks engage in long-range interactions. (B) Such a transition can also be observed in average ($\log_2$ (Observed over Expected)) off-diagonal plots between early origins. For higher J_f, we observe a distinct pattern, which reflects the interactions between the 4 forks (4 loops around the inter-origin 0 position). As J_f increases and larger aggregates are formed with higher probability, the intensity increases. *(Continue in the next page)*

Fig. S17: *(Continued)* (C) Experimental normalized maps in the same genomic region plotted in (A) of replicating cells in early S-phase (WT and Scc1-depleted). No distinct inter-origin enrichment is detected *in vivo*. (D) Same as in (B) but for experimental data. On average off-diagonal plots, we do not detect significant inter-origin enrichment in WT or Scc1-depleted cells when compared to the control without replication (early S-phase, Cdc45-depleted).

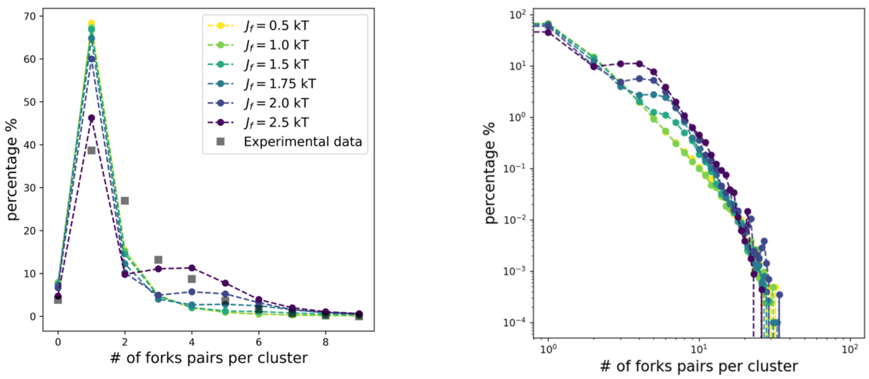

Fig. S18: **Detection of RFi for increasing strength J_f of the non-specific interaction.** Left: percentage of clusters containing a given number of fork pairs as a function of non-specific fork interaction in the interacting sister-fork case ($J_{sister} = 100$ kT). Gray squares indicate the experimental estimate by Saner *et al.*. Simulated data in early-S (between $t = 2$ min and 30 sec and $t = 3$ min and 45 sec) was analyzed to strictly obtain an even number of forks per cluster (see Methods). Right: Same distributions but in logarithmic scale and including the probability for higher number of forks per cluster. No J_f value seems to drastically improve our comparison. The main effect when increasing energy is a higher probability of stabilizing large clusters.

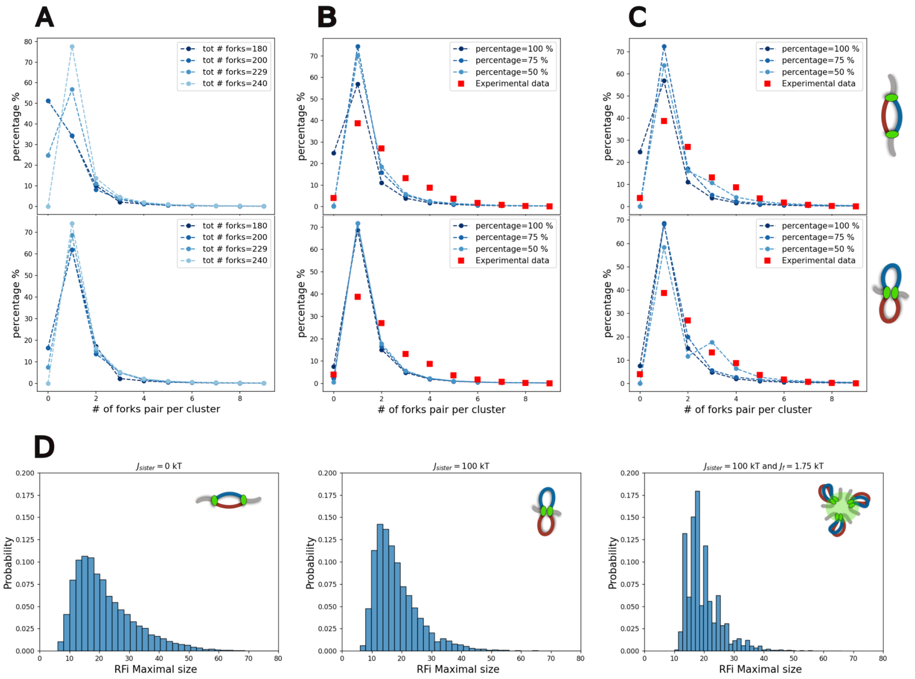

Fig. S19: **Possible confounding factors in RFi detection**. As in Fig. 5D in the main text, distributions are computed with a resolution $\theta = 125$ nm. (A-C) Top and bottom panels correspond to simulations without and with sister-forks interactions respectively. (A) Effect of the total number of forks used to infer RFi sizes. When compared to the distribution computed with the exact average number of forks (299), we demonstrate how in both scenarios small underestimation or overestimation can quantitatively change the distributions. For example, only 20 more forks are sufficient to eliminate the detection of 0-sized clusters (usually coming from isolated single-forks). (B,C) Effects on the distributions of potentially undetected small clusters. Isolated clusters of size 1(B) or 1 and 2(C) are included in the association matrix $A_{i,j}$ at a given percentage. Again, quantitative differences and increased detection of larger clusters are observed. However, none of these changes can fully account for the inconsistency with the experimental data. (D) Distributions of the largest components (RFi with most forks) of each simulation in the 3 scenarios discussed in the main text (Fig. 5D). Very large clusters containing ~ 10 forks are consistently detected. This observation is consistent with the fact that $A_{i,j}$ is often characterized by one larger well-connected cluster and multiple isolated single or sister-forks. For this reason, the probability of having smaller clusters is much higher than intermediate ones (2,3,4 forks pairs), leading to the inconsistency with the experimental estimate.

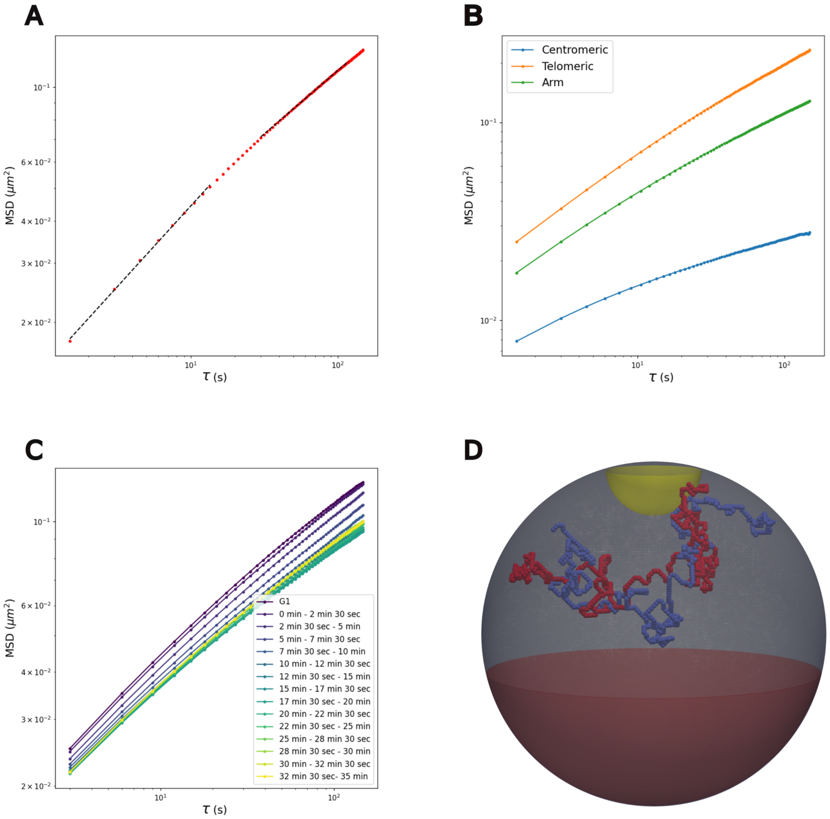

Fig. S20: ***MSD* of chromosomes undergoing replication.** (A) Mean Squared Displacement (MSD) in G1 (red dots), averaged across the full genome and all the simulated trajectories. The two dashed lines at shorter ($<$ 20 sec) and longer τ ($>$ 30 sec) indicates the regime where the MSD scales as $\sim$ 0.5 and $\sim$ 0.3 respectively. (B) MSD in G1 for distinct genomic regions: centromeric monomers (distance $<$ 20 kb from the centromere) (blue), telomeric monomers (distance $<$ 20 kb from chain-ends) (orange), remaining monomers (green). As expected, centromeres diffuse more slowly due to the strong confinement around SPB. Conversely, monomers at proximity of chain-ends (telomeres) diffuse faster. In fact, despite the attachment to the NE, the ends can fluctuate within a distance of 50 nm, likely minimizing its impact on dynamics. *(Continue in next page)*

Fig. S20: *(Continued)* (C) MSD for various time windows in S-phase and in the case of non-interacting sister-forks. The curves correspond to the average across the full genome and among all the simulated trajectories. The data correspond to those discussed in Fig. 6A in the main text while including more time-points. (D) Example of chromosome 8 following replication. The two SCs appear catenated/intertwined as described in our previous study [5].

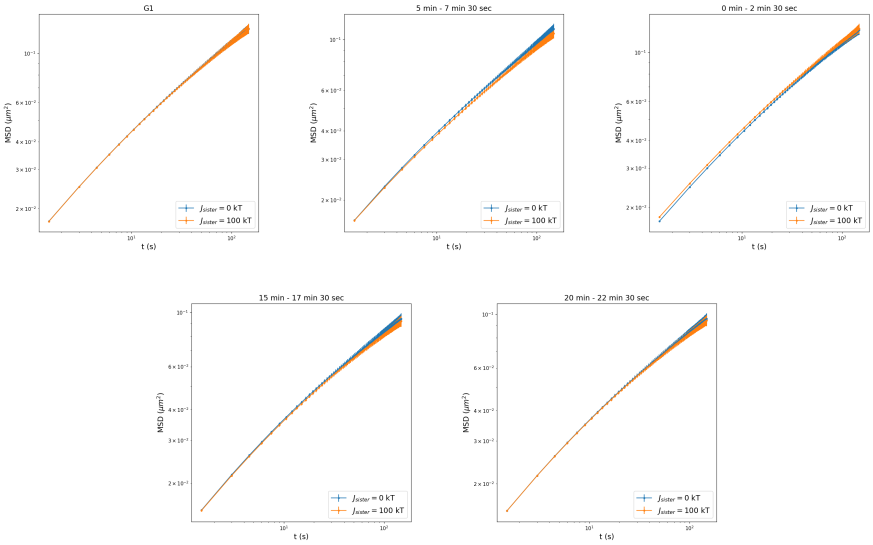

Fig. S21: **Comparison of the overall *MSD* in the two scenarios of sister-forks association**. Each panel corresponds to a different time window used for the computation. The MSD in time does not vary significantly due to the absence (blue curves) or presence (orange curves) of sister-forks interactions.

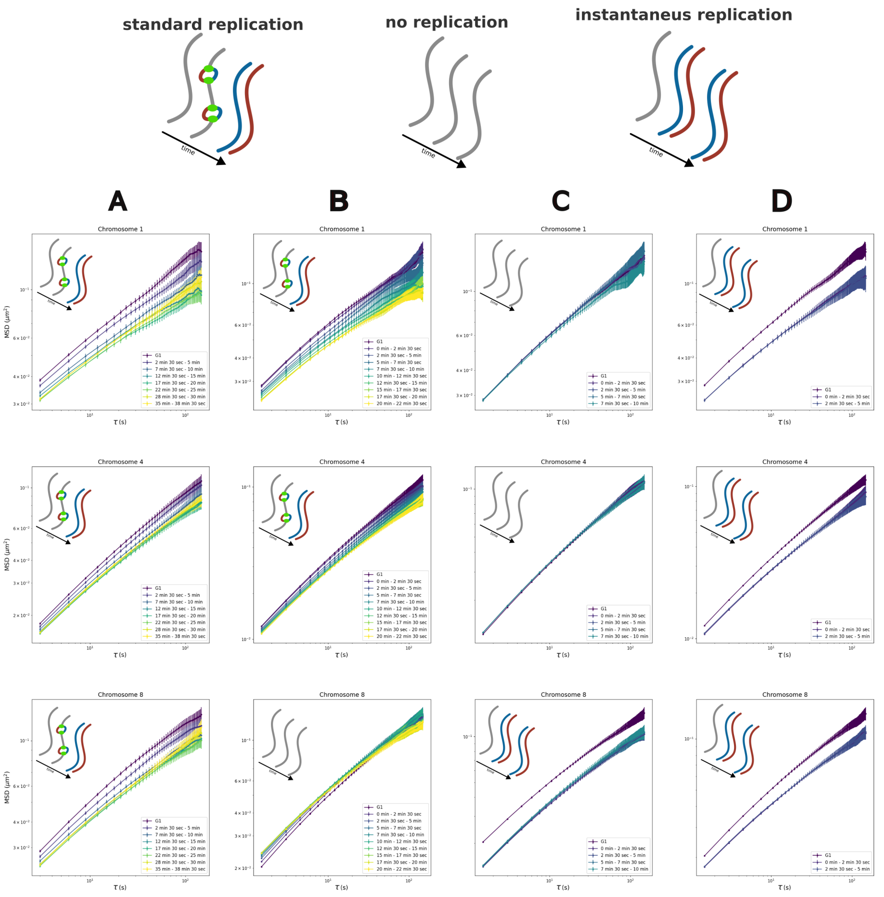

Fig. S22: **Effect of replication-driven catenation on MSD.** Mean Squared Displacement (MSD) for different models when normal replication is perturbed, measured for individual chromosomes. A short (chromosomes 1), long (chromosome 4) and middle (chromosome 8) chains are selected as examples. (Top) Schemes of the replication status of the chromosomes during the simulation. Standard replication: chromosomes are duplicated according to the regular 1D dynamics described in the main text. No replication: origins assigned to the chromosome cannot be fired and SCs are not synthesized. Instantaneous replication: the two SCs are duplicated very fast (less than one simulation frame) one on top of each other resulting in intertwined structures [5]. *(Continue in the next page)*

Fig. S22: (*Continued)* (A) WT-like simulations (Same data used for Fig. S20C and 6A in main text) with standard replication. (B) Model where Chromosome 8 is not replicated (see Methods). In this scenario the time evolution of density is comparable to the WT case. At large times, only the dynamics of chromosome 8 (without catenations) is comparable to its G1 counterpart. (C) Model where only chromosome 8 is instantaneously replicated (see Methods). In this scenario the overall volumic density remains comparable to the one in G1, while, due to replication-driven catenation, chromosome 8 MSD is reduced. (D) Model where the whole genome is instantaneously replicated (see Methods). For all three chromosomes, we observe a decrease in dynamics which is therefore not dependent on the presence the forks in the system.

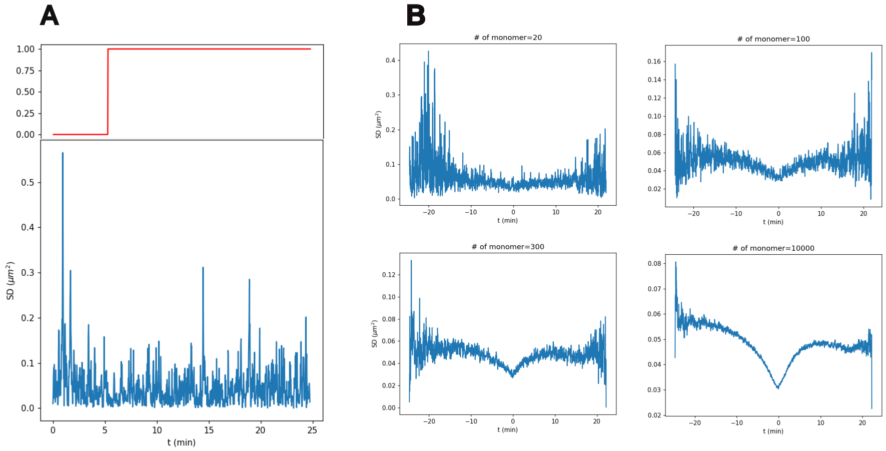

Fig. S23: **Squared displacement SD around the replication time.** (A) $SD(t, \tau = 15\ sec)$ of a single simulation for an individual monomer. At the top, we show the time evolution of the replication status of the monomer, which, around $t = 5$ min goes to 1 (monomer has been duplicated). Fluctuations in SD are too big to observe any effect of replication from a single trajectory. (B) $SD(t, \tau = 15\ sec)$ of a single simulation when SD is averaged among an increasing number of monomers. In particular, a random set of genomic positions were sampled to compute the average. Our analysis suggests that the required number of labeled loci for an individual trajectory (or cells in an experiment) is on the order of 100s.

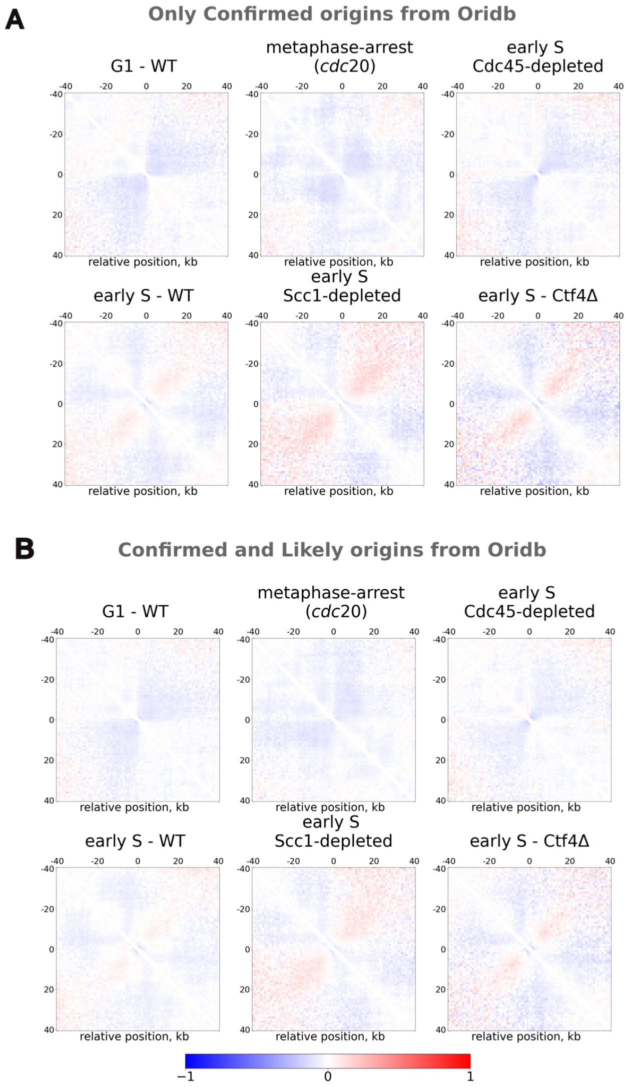

Fig. S24: **Aggregate plots around ARS.** Average normalized ($\log_2$ Observed over Expected) *in vivo* contact maps around (A) “Confirmed” (blue bands in Fig. S3C) and (B) “Confirmed” and “Likely” ARS (red lines and green in Fig. S3C). The computation was performed for all the mutant strains discussed in the main text with the same conclusion (See Fig. 3A for more information). Consistent with the high number of ARS in the Oridb database which do not correspond to inferred early origins (see Fig. S6C), the signal appears in both cases much lower than in Fig. 3A where, thanks to the predictive power of the IPLS, we could restrict the average to origins more likely to be fired in early S-phase.

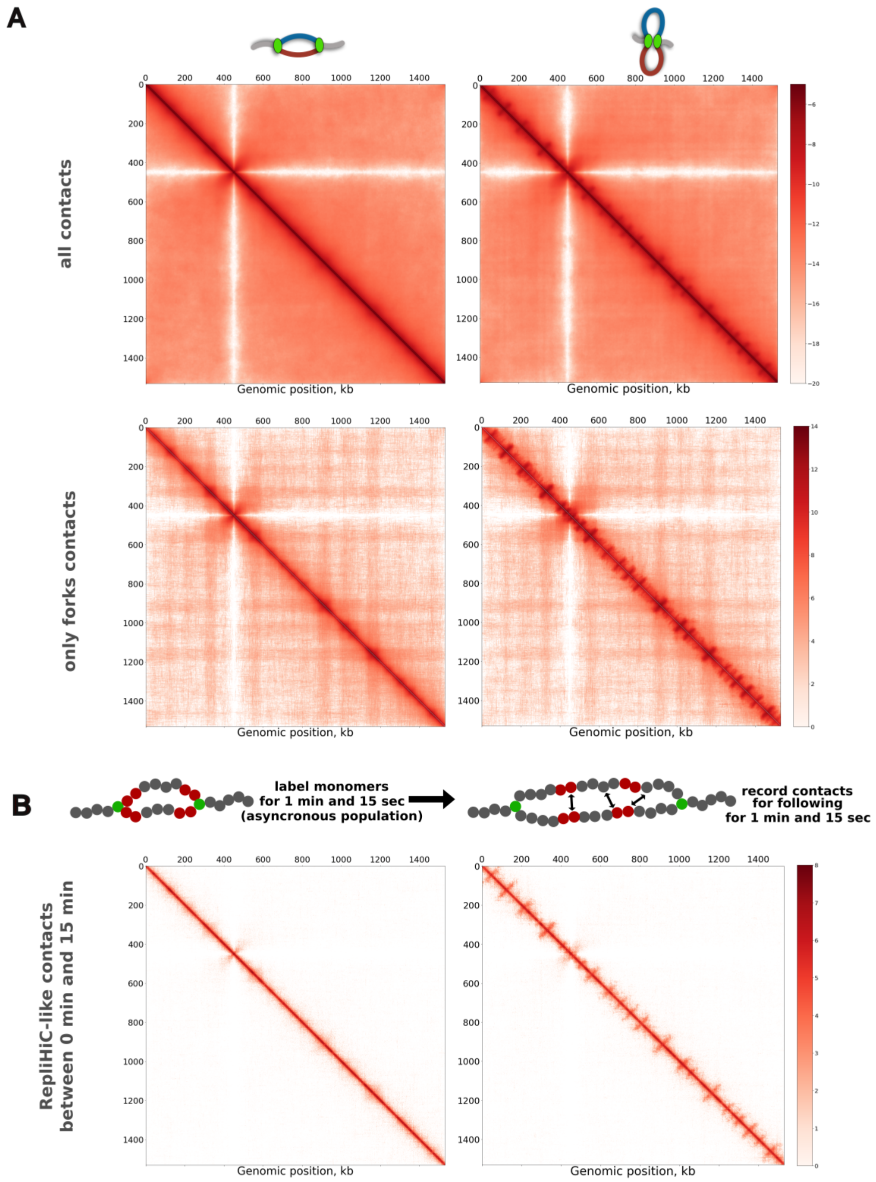

Fig. S25: **Simulating variant Hi-C techniques.** *In silico* contact maps of chromosome 4 for the non-interacting (left) and interacting (right) cases, under different variations of the Hi-C technique, where only a subset of contacts is retained. (A) Top: Standard Hi-C-like predictions where all contacts are used. Bottom: HiChIP-like predictions where only contacts implicating at least one replication fork are included. *(Continues in next page)*

Fig. S25: *(Continued)* For both cases, we used a time window spanning 1 minute and 15 seconds of G1 and the first 11 minutes and 30 seconds of S-phase. As expected, fountains are highly enhanced in HiChIP-like maps in the interacting scenario, while, in the non-interacting case, only a minor enrichment around early origins (due to the homogeneous compaction described in the main text) is observed within such a large time window. (B) Repli-Hi-C-like contact maps. To mimic the Repli-Hi-C protocol developed by Liu *et al.* in [6], where only contacts between nascent DNA loci are retained, we followed the approach illustrated in the scheme at the top: we labeled newly replicated monomers during 1 minute and 15 seconds, starting from a random time point during the simulated S-phase to simulate an asynchronous population. Only pair-wise contacts in which at least one of the two monomers is labeled are included. In this case, we assumed that each labeled monomer can establish only one contact. Interestingly, we recover clear fountains in the interacting case, as observed in mammalian cells in [6], while the non-interacting case shows only on-diagonal enrichment.

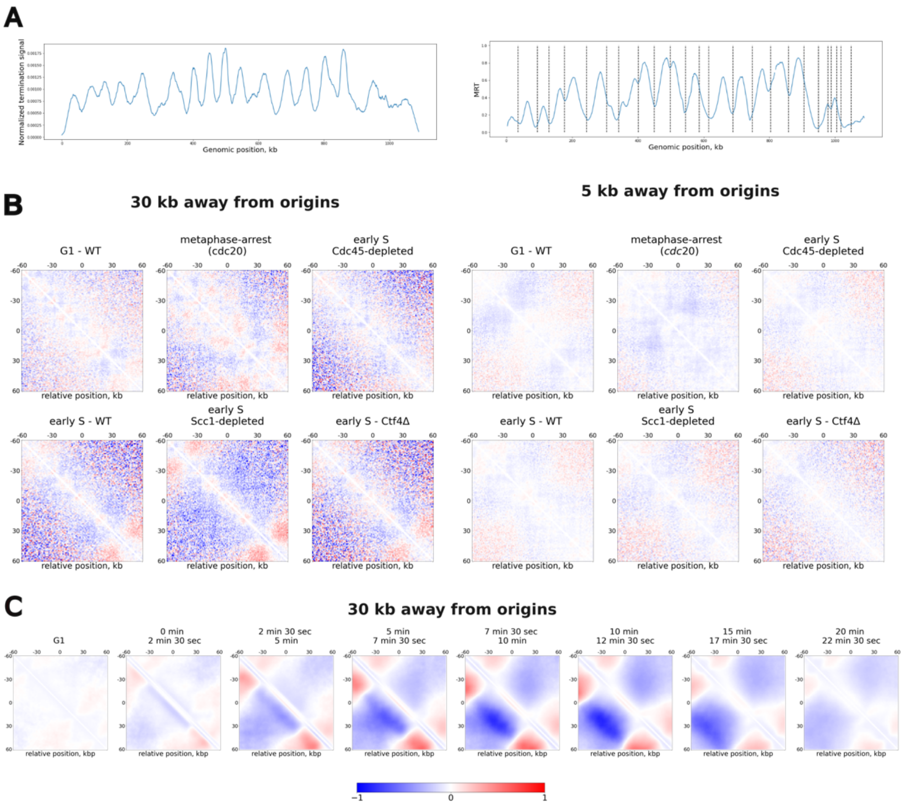

Fig. S26: **Hi-C signal at termination sites.** (A) Example of termination sites for chromosome 4. Starting from a population of $\sim$ 1000 simulated trajectories, we create a distribution for termination events (panel on the left). Such events can be easily detected in our formalism when two convergent forks meet (bubbles merging, see Methods). After smoothing (20 kb window) we apply the scipy function $find\_peaks$ [7] with a prominence of 0.5 and minimum distance of 4 kb to detect termination sites. On the right we plot the experimental MRT [4] of the same chromosome with vertical lines at the detected termination zones. (B) Average normalized ($\log_2$ Observed over Expected) *in vivo* contact maps around termination sites removing sites located at a distance less than 5 (left) and 30 kb (right) from origins to filter the sites that may act both as origin and termination sites. We do not observe any clear signal around the termination sites during S-phase. (C) Average normalized ($\log_2$ Observed over Expected) *in silico* contact maps at different time steps around termination sites located at a distance less than 30 kb from origins.

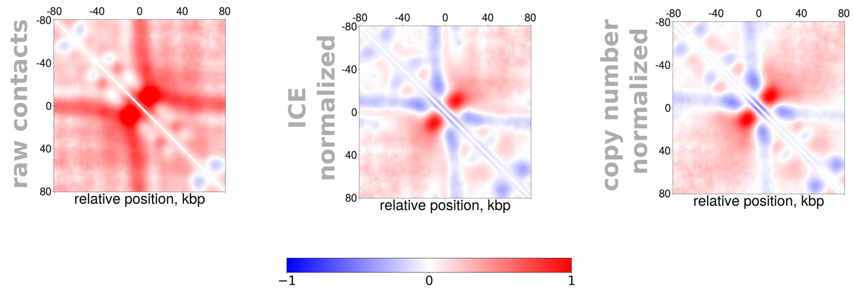

Fig. S27: **Effect of ICE normalization on replicating polymers.** When computing raw contact maps of two not-fully replicated sister chromatids, some regions can present strong contact enrichments, not driven by more frequent contacts but rather by an increase in copy number during replication. To test if the ICE balancing method correct for that, we adapt the parameters of the underlying 1D replication dynamics [3] and simulate the single chromosome 4 in a box with periodic boundary conditions and with interacting sister-forks. (Left) Computing raw contact maps in early S, we observe a strong contact enrichment (red cross) that corresponds to the average replicon size and is related to the changes in copy-number (similarly to what is shown in Fig. S9A). (Middle) Corresponding map after ICE normalization. (Right) Map exactly corrected for the copy-number, i.e., each raw bin $M_{i,j}^{raw}$ of the left panel is divided by $(Cn_j \cdot Cn_i)$ where Cn_i is the average copy number of monomer i. Remarkably little difference is found between ICE- and copy-number-normalized maps. Such reliability of using standard normalization methods on replicating cells is quite crucial when comparing to the experiments. Indeed, normalizing through the average copy number Cn_i (therefore replication status) of a given genomic position i is easily accessible *in silico* but it does not have a direct counterpart for *in vivo* data.

Supplementary Videos

- **Additional file 3: Video S1**:
  `https://youtu.be/yjj7ZIhEZC0`
  Example of a full genome simulation with chromosomes undergoing replication with sister-forks interactions. Unreplicated DNA is shown with gray beads, while newly synthesized SCs in blue and red. Green beads pinpoint replication forks. The red region within the sphere indicates nucleolus and is inaccessible to other monomers. The yellow shell indicates the surface where centromeres are attached (SPB)

- **Additional file 4: Video S2**:
  `https://youtu.be/5VMm6JvkvtY`
  Same as Additional file 3: Video S1 but in the case of non-interacting forks and for a longer simulation which covers all the S-phase.

- **Additional file 5: Video S3**:
  `https://youtu.be/Zggx52Np6OE`
  (Right) Forks in the nucleus in the presence of sister-fork interactions (same simulation used for Additional file 3: Video S1). (Left) Corresponding distribution as a function of the distance r from the SPB in time. The distribution is normalized by the corresponding volume of the slice: $V(r + dr) - V(r)$ where $V(r) = (3R-)r^2\pi/3$. The vertical line corresponds to the position of the yellow spherical shell on the Right.

- **Additional file 6: Video S4**:
  `https://youtu.be/ng3g2kbbub8`% Same as Additional file 5: Video S3 but in the case of non-interacting forks and for a longer simulation which covers all the S-phase.

References

1. Hediger, F., Neumann, F. R., Van Houwe, G., Dubrana, K. & Gasser, S. M. Live imaging of telomeres: yKu and Sir proteins define redundant telomere-anchoring pathways in yeast. *Current biology* **12,** 2076–2089 (2002).
2. Siow, C. C., Nieduszynska, S. R., Müller, C. A. & Nieduszynski, C. A. OriDB, the DNA replication origin database updated and extended. *Nucleic acids research* **40,** D682–D686 (2012).
3. Arbona, J.-M. *et al.* Neural network and kinetic modelling of human genome replication reveal replication origin locations and strengths. *PLOS Computational Biology* **19,** e1011138 (2023).
4. Müller, C. A. *et al.* The dynamics of genome replication using deep sequencing. *Nucleic acids research* **42,** e3–e3 (2014).
5. D'Asaro, D., Tortora, M. M., Vaillant, C., Arbona, J.-M. & Jost, D. DNA replication and polymer chain duplication reshape the genome in space and time. *Physical Review X* **14,** 041020 (2024).
6. Liu, Y. *et al.* Fork coupling directs DNA replication elongation and termination. *Science* **383,** 1215–1222 (2024).
7. Virtanen, P. *et al.* SciPy 1.0: fundamental algorithms for scientific computing in Python. *Nature methods* **17,** 261–272 (2020).
